# Data Sharing by Scientists: Practices and Perceptions

**DOI:** 10.1371/journal.pone.0021101

**Published:** 2011-06-29

**Authors:** Carol Tenopir, Suzie Allard, Kimberly Douglass, Arsev Umur Aydinoglu, Lei Wu, Eleanor Read, Maribeth Manoff, Mike Frame

**Affiliations:** 1 School of Information Sciences, University of Tennessee, Knoxville, Tennessee, United States of America; 2 University of Tennessee Libraries, University of Tennessee, Knoxville, Tennessee, United States of America; 3 Center for Biological Informatics, United States Geological Survey, Oak Ridge, Tennessee, United States of America; Science and Technology Facilities Council, United Kingdom

## Abstract

**Background:**

Scientific research in the 21st century is more data intensive and collaborative than in the past. It is important to study the data practices of researchers – data accessibility, discovery, re-use, preservation and, particularly, data sharing. Data sharing is a valuable part of the scientific method allowing for verification of results and extending research from prior results.

**Methodology/Principal Findings:**

A total of 1329 scientists participated in this survey exploring current data sharing practices and perceptions of the barriers and enablers of data sharing. Scientists do not make their data electronically available to others for various reasons, including insufficient time and lack of funding. Most respondents are satisfied with their current processes for the initial and short-term parts of the data or research lifecycle (collecting their research data; searching for, describing or cataloging, analyzing, and short-term storage of their data) but are not satisfied with long-term data preservation. Many organizations do not provide support to their researchers for data management both in the short- and long-term. If certain conditions are met (such as formal citation and sharing reprints) respondents agree they are willing to share their data. There are also significant differences and approaches in data management practices based on primary funding agency, subject discipline, age, work focus, and world region.

**Conclusions/Significance:**

Barriers to effective data sharing and preservation are deeply rooted in the practices and culture of the research process as well as the researchers themselves. New mandates for data management plans from NSF and other federal agencies and world-wide attention to the need to share and preserve data could lead to changes. Large scale programs, such as the NSF-sponsored DataNET (including projects like DataONE) will both bring attention and resources to the issue and make it easier for scientists to apply sound data management principles.

## Introduction

Data are the infrastructure of science. Sound data are critical as they form the basis for good scientific decisions, wise management and use of resources, and informed decision-making. Moreover, “science is becoming data intensive and collaborative” [1]. The amount of data collected, analyzed, re-analyzed, and stored has increased enormously due to developments in computational simulation and modeling, automated data acquisition, and communication technologies [2]. Following the previous research paradigms (experimental, theoretical, and computational), this new era has been called “the fourth paradigm: data-intensive scientific discovery” where “all of the science literature is online, all of the science data is online, and they interoperate with each other” [3]. Digital data are not only the outputs of research but provide inputs to new hypotheses, enabling new scientific insights and driving innovation [4].

As science becomes more data intensive and collaborative, data sharing becomes more important. Data sharing includes the deposition and preservation of data; however, it is primarily associated with providing access for use and reuse of data. Data sharing has many advantages, including:

- re-analysis of data helps verify results data, which is a key part of the scientific process;
- different interpretations or approaches to existing data contribute to scientific progress –especially in an interdisciplinary setting;
- well-managed, long-term preservation helps retain data integrity;
- when data is available, (re-)collection of data is minimized; thus, use of resources is optimized;
- data availability provides safeguards against misconduct related to data fabrication and falsification;
- replication studies serve as training tools for new generations of researchers [5] [6] [7]

Additionally, researchers, data managers and publishers in the PARSE survey overwhelmingly agreed that public funding was the most important reason for data preservation. Nearly all (98%) of participants agreed that if research is publicly funded, the results should become public property and therefore properly preserved [8].

This article reports the results of a survey of scientists' current data sharing practices and their perceptions of the barriers and enablers of data sharing. The survey was conducted by the research team of the National Science Foundation-funded DataONE project. DataNet supports short- and long-term data management and open access to data. DataONE is one of the initially funded NSF DataNet partners. DataONE is a large scale collaboration to develop an organization that supports the full information lifecycle of biological, ecological, and environmental data and tools to be used by researchers, educators, students, decision-makers and the general public. DataONE “will ensure the preservation and access to multi-scale, multi-discipline, and multi-national science data” [9] by developing a strong cyberinfrastructure and community engagement programs.

DataONE will (i) provide coordinated access to current data collections; (ii) create a new global cyberinfrastructure that contains both biological and environmental data coming from different resources (research networks, environmental observatories, individual scientists, and citizen scientists); and (iii) change the science culture and institutions by providing education and training, engaging citizens in science, and building global communities of practice. In order to facilitate change of the science culture through cyberinfrastructure for data, it is necessary to first understand the culture of modern science and the role of data in it.

### Data Sharing

**Figure 1:**
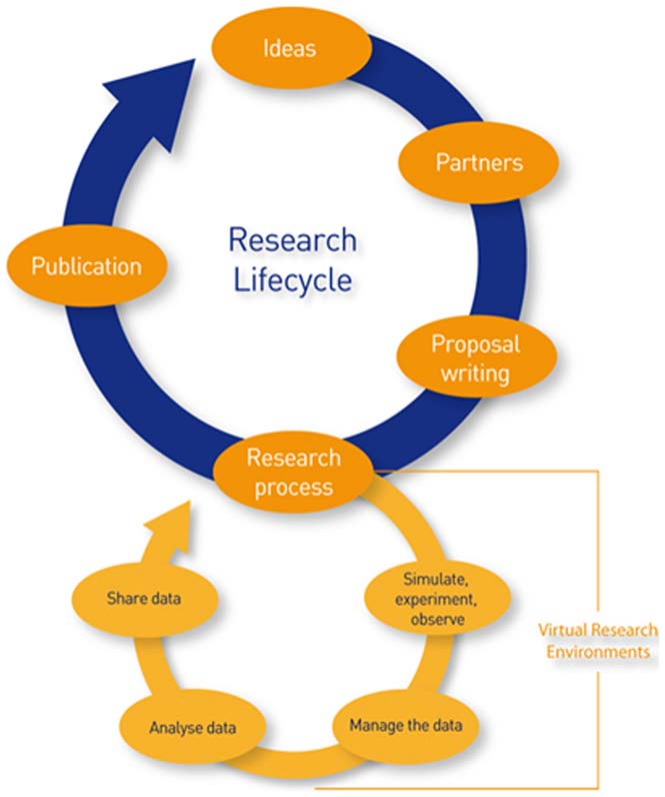
Joint Information Systems Committee (JISC), Stages of the research and data lifecycle.

Encouraging data sharing and reuse begins with good data practices in all phases of the data lifecycle such as generating and collecting the data, managing the data, analyzing the data, and sharing it. However, the data lifecycle cannot be considered independently from research lifecycle [10], as data are an indispensible element of scientific research. (See Figure 1.)

The specific costs of handling supplementary materials such as datasets are not well documented. In a recent survey, only author fees and journal subscription fees were mentioned as current funding sources for supplementary materials in journals. Participants in the survey suggested other potential sources for funding, in particular government funding, support from learned societies, and publishers [11].

### Data Sharing/Withholding Practices

Data sharing is important. According to a study done by Publishing Research Consortium (PRC) in 2010 with 3823 respondents, access to datasets, data models, and algorithms & programs was ranked important or highly important; however, only 38% of them felt that they were easily accessible [12]. In addition, it was the lowest among the other information types (some of them were research articles in journals, reference works, technical information, patent information, etc.). Several previous surveys have explored the benefits and barriers of sharing data [13] and the extent to which researchers share or withhold data. Results seem to suggest that current sharing practices are minimal, although the amount of data sharing varies among different fields. Some journals have specific guidelines which require authors to share their data with other researchers. However, the extent to which these guidelines are carried out remains largely untested. Savage and Vickers requested data from ten researchers who had published articles in PLoS journals, which have specific data sharing policies. Only one author sent an original dataset [14]. Although drawn from a small sample of researchers, these results strongly suggest that journal policies which require data sharing do not necessarily lead authors to make their datasets readily available to other researchers. The amount of data sharing or data hoarding also appears to vary according to the researcher's subject discipline.

Researchers who choose to withhold datasets often have specific reasons for doing so. Savage and Vickers noted reasons that include concerns about patient privacy (for medical fields), concerns about future publishing opportunities, and the desire to retain exclusive rights to data that had taken many years to produce [14]. In Campbell's study of data sharing in genetics, the top reasons cited for withholding data were the amount of effort involved in accessing and sharing datasets and the protection of a colleague's or their own ability to publish [15]. The decision to share or withhold data is often dependent upon the point of time in the publishing process at which the request is made. Campbell (2003) reported that nearly all (98.7%) of the technology transfer officers surveyed agreed that academic scientists should freely share data with other scientists after publication, while only 30.5% agreed that scientists should share data and materials before publication. The vast majority also believed that scientists should be more careful when sharing data with industry than with other academics [15]. The PARSE Insight survey indicated that researchers who are reluctant to share data with others reported major concerns with legal issues, misuse of data, and incompatible data types [8]. In a survey of geneticists and other life scientists, Campbell et al., found that withholding data may be more common in genetics and related fields. Reasons may include the increased scientific competitiveness of the field, as well as the opportunities for commercial applications. Respondents of the survey estimated that ten percent of their requests for information from other researchers in the field were denied [16]. These results do not include other data practices which may also negatively affect the progress of science, such as significant delays in the fulfillment of requests, refusals to publicly present research findings, and the failure to discuss research with others [16]. Disciplines or subdisciplines have their own culture of data-sharing. Some do better (geophysics, biodiversity, and astronomy) than others [17].

### Individual Choice vs. Institutional Policies

The extent to which researchers share or withhold data is not primarily an individual choice. Underlying policies and practices have great influence on encouraging or inhibiting data sharing. Several researchers who failed to share their data in the study by Savage and Vickers, et al., claimed that it would take too much work to provide raw data. The authors came to the conclusion that researchers often fail to develop clear, well-annotated datasets to accompany their research (i.e., metadata), and may lose access and understanding of the original dataset over time. Vickers, et al. believe that a policy that would require authors to submit datasets to journals or public repositories at the time of publication would help to prevent this occurrence [11]. PARSE Insight, a project concerned with the preservation of digital information in research, reported from a survey of data managers that 64% claimed their organizations had policies and procedures in place to determine what kinds of data are accepted for storage and preservation, with specific policies for the time frame and method of submission. Though this number constitutes a majority, 32% reported a lack of such policies or procedures [8].

Policies and procedures sometimes serve as an active rather than passive barrier to data sharing. Campbell et al. (2003) reported that government agencies often have strict policies about secrecy for some publicly funded research. In a survey of 79 technology transfer officers in American universities, 93% reported that their institution had a formal policy that required researchers to file an invention disclosure before seeking to commercialize research results. About one-half of the participants reported institutional policies that prohibited the dissemination of biomaterials without a material transfer agreement, which have become so complex and demanding that they inhibit sharing [15].

Increasing the efficiency of current data practices in a world of increased data challenges requires a new comprehensive approach to data policy and practice. This approach would seek to avoid data loss, data deluge, poor data practices, scattered data, etc., and thus make better use of (public) funds and resources. NSF recently took action by announcing that all proposals to NSF involving data collection must include a data management plan [1] so that “digital data are routinely deposited in well-documented form, are regularly and easily consulted and analyzed by specialist and non-specialist alike, are openly accessible while suitably protected, and are reliably preserved” [18]. Similarly, the European Commission invited its member states to develop policies to implement access, dissemination, and preservation for scientific knowledge and data [5] [19].

### Data Sharing Tools

The life sciences and other fields rely on observational data and the situation is growing increasingly complex as data is used for advanced modeling which also creates new datasets. Paton, in an analysis of the management of experimental data in the life sciences, noted that several standards bodies are beginning to make use of common foundation models, most notably the FuGE (Functional Genomics Experiment), which provides consistency in practice among scientific communities. Also, minimum information guidelines already exist in the systems biology community. For example, the widely used Systems Biology Markup Language provides a format by which models can be shared. In addition, a Systems Biology Ontology is under development. These standards have received consistent support from software tools, public repositories, and journals. Information guidelines for other disciplines are not yet as structured. Many disciplines, particularly experimentally-based disciplines, lack accepted standards for their research activities [20]. The Dryad project, a digital repository for the publication of scientific data, reported in a 2010 survey that of 12 journal publishers, several journals require authors to deposit some form of data into existing databases. However, few policies specifically address metadata, long-term preservation, or access for supplementary materials [11].

### Supporting Cyberinfrastructure

The development of cyberinfrastructure will play a major role in the future sharing of data. Participants in the PARSE survey named lack of sustainable hardware, software, and support of computer environment as the most important threats to digital preservation. The majority of researchers, data managers, and publishers who participated in the survey believed that an international infrastructure for data preservation should be built [8]. Data needs to be stored and organized in a way that will allow researchers to access, share, and analyze the material. The Dryad Repository is attempting to address this need by providing users with the ability to access supplementary materials using search engines and perform advanced searches within datasets. Participants in their survey reported that the possibility of assigning Digital Object Identifiers (DOI's) to data in order to cite material was particularly appealing [11]. Paton notes that few well established data integration infrastructures exist in the life sciences, and significant software development or tailoring activities are often carried out in-house in individual laboratories. The development of data standards may provide a foundation for cross-community collaboration in format and ontology development, making it much easier for laboratories to manage, integrate, and analyze data [20].

## Methods

This international survey of scientists' data practices and perceptions has identified what scientists in many fields are doing now in terms of data collection, data use, storage, and reuse. In addition, the survey addresses questions related to perceptions of barriers that may hinder data sharing and reuse. The solutions to increased data sharing will be the result of overcoming these barriers, including those relating to perceptions and motivations of scientists, availability of appropriate tools, and a cyberinfrastructure that makes sharing possible. DataONE hopes to eventually help provide viable solutions.

### Methodology

The University of Tennessee Human Subjects Institutional Review Board approved this study as an online survey with the anonymity of respondents protected. No identifying questions were asked and this paper reports the findings in the aggregate. The survey was open for responses from October 27, 2009 to July 31, 2010. Initially, the investigators used a snowball sampling method. Investigators sent an email cover letter to DataONE team members (about 35 individuals throughout the world, but primarily in the United States). The letter contained a survey link that members distributed to others who could champion the effort to distribute the link inside academic and research organizations. Federal agencies that manage and produce large amounts of research data were also targeted for participation. In an academic setting, this champion could be a dean or department-chairperson who would circulate the link to faculty, lecturers, post doctoral research associates, graduate students, and undergraduate students. At a research facility, this champion could be a research director who would circulate the link to researchers in the organization. A number of DataONE team members acted as champions inside their respective organizations.

In March 2010 investigators began targeting the survey to universities in states with the lowest number of responses to date. This helped increase the breadth of the sample. To increase international response, surveys were sent by an academic publisher to its database of over 7,000 previous authors. Given the electronic distribution method, there is no way of knowing the total number of survey link recipients. Ultimately, 1329 respondents answered at least one question. It is not unreasonable to estimate that the survey instrument reached 15,000 people, in which case the response rate is approximately 9%.

### Research Instrument

The survey instrument consisted of two sections: questions about demographics and questions about scientists' relationship with data. A complete version is found in the Appendix S1.

Subjects were asked their age and gender, the sector they work in, their subject discipline, their professional position, their primary funding agency, and their primary country of employment. If employed in the U.S., respondents were also asked to indicate their state. In order to measure differences in data practices between respondents who focus most of their work time on research and those who spend more time on teaching, administration, or other duties, respondents were asked to estimate the percent of their work time spent on various activities.

To discover respondents' data practices, perceptions, and attitudes, several different types of questions were asked. Respondents were asked to what degree they agree or disagree with a series of statements, in addition to some yes/no and open-ended questions. Each question or statement explored a different aspect of data issues: the type of data used; whether they are available to others (if yes, how and if no, why); collection and use of research data; the relationship between their organization and their data; the use of data across their respective research field; views on data sharing and fair exchange of data; responsibility for their data; and the relationship between their funding agency and their data. Yes/No questions were about the conditions for a fair exchange for the use of data (respondents using other people's data and other people using respondents' data). Such conditions include receiving co-authorship, having the opportunity to collaborate, recovering costs of data acquisition, retrieval or provision, obtaining legal permission, etc. There were two open-ended questions. One was about additional approvals for others to access data the provider's data and the other asked for additional comments, questions, or suggestions about use of data. Also, when the choices that were provided in the answer did not accurately represent the respondent's condition, the respondent was asked to describe the condition in an “other” category.

## Results and Discussion

This findings section begins by providing an overview of respondent characteristics. It then provides a detailed look at several key data practice concepts addressed in the study – data use, data practices, data management support and policies, data reuse and, most importantly, data sharing. The final section of the findings looks at two key concepts, data reuse and data sharing by different demographic groups, including primary funding source, subject discipline, age of researcher, primary activity of researcher, and location of researcher.

### Demographics of Respondents

Seventy-nine percent (79%) of respondents indicated they spend 50% or more of their time on research which were designated as “research intensive” respondents. Twenty-one percent spend 50% or more of their time on teaching which were designated as “teaching intensive” respondents. Most respondents are academics who split their time between research, teaching, administration, and other activities. The majority of respondents (80%) work in academic institutions, 13% work in government, and the rest work in commercial, non-profit or other settings (see Table 1).

**Table 1:** Primary work sector.

	Frequency	Percent
Academic	1058	80.5
Government	167	12.7
Commercial	34	2.6
Non-profit	35	2.7
Other	21	1.6
**Total**	**1315**	**100.0**

Since most of the respondents come from academic institutions, it is not surprising that 47% of respondents hold the academic titles (or equivalent) of assistant professor (10.5%, n = 137), associate professor (14.3%, n = 187) or professor (22.2%, n = 291). The next most common position is researcher (21.1%, n = 276) or student (graduate student 13.5%, n = 177).

Respondents represent a variety of science and social science subject disciplines, with most from the DataONE target disciplines, environmental sciences and ecology (36%, n = 475). The breakdown of respondents by disciplines is provided in Table 2.

**Table 2:** Subject discipline.

	Frequency	Percent
environmental sciences & ecology	475	36.1
social sciences	204	15.5
biology	181	13.7
physical sciences	158	12.0
computer science/engineering	118	9.0
other	98	7.4
atmospheric science	52	3.9
medicine	31	2.4
**Total**	**1317**	**100.0**

Nearly three quarters (73%, n = 930) of the respondents are from North America, 15% (n = 188) are from Europe, and 7.3% (n = 94) are from Asia/Oceania. Of the U.S. respondents, 36% (n = 301) are from the South, 26% (n = 220) are from the Midwest, 25% (n = 207) are from the West, and 14% (n = 121) are from the Northeast.

Of the respondents who provided their ages, 38% (n = 453) are between 20 and 39 years old, 30% (n = 359) are between 40 and 50 years old, and 33% (n = 393) are over 50, for a mean age of 44.8 Two thirds of the respondents are male and one third are female.

### Current Data Practices

It is important to note that the survey predated NSF's requirement for data management plans. Beginning on January 18, 2011 proposals submitted to NSF must include a data management plan. At the time of the survey, NSF did not require such plans for its funded projects. When respondents were asked whether their primary funding agency requires them to provide a data management plan, more than half (55%) reported no, 29% yes, and 16% said they do not know.

#### Data Use

Some respondents currently access data from local, national or international networks. The most commonly used are organization-specific systems (39%) and the Long-term Ecological Research Network (LTER) (32%). Respondents could select more than one source (see Table 3).

**Table 3:** Data access.

	Frequency	Percent
An organization-specific system	351	38.5%
Long-tem Ecological Research Network	292	32.1%
Other data access	246	27.0%
A Distributed Active-Archive Center	173	19.0%
A Global Biodiversity Information Facility	73	8.0%
National Biological Information Infrastructure	70	7.7%
National Ecological Observatory Network	64	7.0%
International Long-term Ecological Research Network	58	6.4%
Taiwan Ecological Research Network	7	.8%
South African Environmental Observation Network	6	.7%

Respondents reported that they use various types of data in their research, including experimental data, observational data, data models, abiotic surveys, and biotic surveys. Many respondents use more than one data type. The responses for the various types of data used are presented in Table 4.

**Table 4:** Data types.

	Responses	Percent
Experimental	711	54.6%
Observational	632	48.5%
Data Models	499	38.3%
Biotic Surveys	446	34.3%
Abiotic Surveys	442	33.9%
Remote-Sensed Abiotic	358	27.5%
Remote-Sensed Biotic	264	20.3%
Social Science Surveys	251	19.3%
Interviews	195	15.0%
Other	80	6.1%

#### Data Practices

A majority of the respondents are satisfied with their current processes for most of the initial and short-term parts of the research and data lifecycle, including collecting their research data, searching for their data, analyzing their data, and short-term storage of their data. A smaller majority say they are satisfied with cataloging or describing their data (59.8% agree strongly or somewhat). However, the satisfaction rate for the process of storing their data beyond the life of the project (long-term) is much lower than the short-term, only 45% versus 73%. More than a third (35%) of the respondents stated that they are dissatisfied with the long-term storage process (see Table 5).

**Table 5:** Data issues.

I am satisfied with the process for,,,	Agree Strongly	Agree Somewhat	Neither Agree Nor Disagree	Disagree Somewhat	Disagree Strongly
… collecting my research data.	410 (31.6%)	626(48.2%)	139 (10.7%)	112 (8.6%)	11 (0.8%)
… searching for my own data.	298 (23.2%)	600 (46.7%)	230 (17.9%)	141 (11%)	16 (1.2%)
… cataloging/describing my data.	226 (18%)	526 (41.8%)	273 (21.7%)	194 (15.4%)	40 (3.2%)
… storing my data during the life of the project (short-term).	376 (29.2%)	559 (43.5%)	189 (14.7%)	143 (11.1%)	19 (1.5%)
… storing my data beyond the life of the project (long-term).	206 (16%)	369 (28.6%)	271 (21%)	334 (25.9%)	111 (8.6%)
… analyzing my data.	383 (29.7%)	598 (46.4%)	177 (13.7%)	118 (9.1%)	14 (1.1%)

Effective data management and use relies on effective tools. A series of questions about satisfaction with tools for all aspects of the data lifecycle reveal some variation in satisfaction (see Table 6). Only about a quarter (26%) of the respondents is satisfied with the tools for preparing metadata, while over 32% are dissatisfied. The large number of respondents who replied that they neither agree nor disagree (42%) could be interpreted in two ways: either they truly are indifferent or they are unsure about what metadata means. There is some reason to believe that the latter is true as nearly half (46%) of the respondents answered “none” to the question “What metadata do you currently use to describe your data?” Forty two percent reported that they are satisfied with the tools for preparing their documentation; however, 31% indicated that they neither agree nor disagree. Clearly, there is room for more effective tools and education as it applies to metadata concepts and principles as a component of data management.

**Table 6:** Data tools.

	Agree Strongly	Agree Somewhat	Neither Agree Nor Disagree	Disagree Somewhat	Disagree Strongly
I am satisfied with the tools for preparing metadata.	75 (6%)	252 (20%)	526 (41.7%)	289 (22.9%)	118 (9.4%)
I am satisfied with the tools for preparing my documentation.	155 (12.1%)	413 (32.3%)	409 (32%)	231 (18.1%)	71 (5.6%)

#### Data management support and policies

Institutions can help or hinder good data management. Policies and assistance with data management across the data lifecycle vary among institutions. While 43% of the respondents agreed that their organization or project has a formal established process for managing data during the life of the project, almost half (47%) of the respondents disagreed with the statement that their organization or project has a formal established process for storing data beyond the life of the project. Only 38% of the respondents reported that they have a formal established process for storing data long-term, while 45% of the respondents replied that their organization provides, to a degree, the necessary tools and technical support for data management during the life of the project (short-term). Only one third (35%) of the respondents are provided with the necessary tools and technical support for long-term data management.

Nearly half (48%) of the respondents reported that their organization or project does not provide the necessary funds to support data management during the life of a research project. More than half (59%) indicated that their organization or the project does not provide training on best practices for data management. Also, 59% of the respondents replied that their organization or project does not provide the necessary funds to support data management beyond the life of the project (see Table 7). Institution and Agency initiatives, including efforts like DataONE, can greatly improve these results.

**Table 7:** Organizational involvement in data issues.

My organization or project…	Agree Strongly	Agree Somewhat	Neither Agree Nor Disagree	Disagree Somewhat	Disagree Strongly
… has a formal established process for managing data during the life of the project (short-term).	221 (17.2%)	330 (25.6%)	183 (14.2%)	257 (20%)	297 (23.1%)
… has a formal established process for storing data beyond the life of the project (long-term).	200 (15.6%)	294 (22.9%)	191 (14.9%)	271 (21.1%)	328 (25.5%)
… provides the necessary tools and technical support for data management during the life of the project (short-term).	192 (15%)	374 (29.2%)	269 (21%)	221 (17.3%)	224 (17.5%)
… provides the necessary tools and technical support for data management beyond the life of the project (long-term).	155 (12.1%)	294 (22.9%)	232 (18%)	204 (23.6%)	301 (23.4%)
… provides training on best practices for data management.	75 (5.9%)	199 (15.5%)	253 (19.8%)	339 (26.5%)	414 (32.3%)
… provides the necessary funds to support data management during the life of a research project (short-term).	115 (9%)	275 (21.4%)	273 (21.3%)	296 (23.1%)	325 (25.3%)
… provides the necessary funds to support data management beyond the life of the project (long-term).	85 (6.6%)	194 (15.1%)	249 (19.4%)	314 (24.4%)	443 (34.5%)

#### Data Reuse

We asked respondents about their views on the use of data across their research field. Note that this measures their perceptions or opinions and does not necessarily completely reflect actual practice. Still, the level of agreement or disagreement with these statements reveals many psychological barriers to good data sharing practice.

Respondents were asked their agreement on a five-point scale to a series of statements (see Table 8). Nearly two thirds (67%) of the respondents agreed that lack of access to data generated by other researchers or institutions is a major impediment to progress in science. Half (50%) of the respondents reported that lack of access to data generated by other researcher or institution has restricted their ability to answer scientific questions. Three quarters (75%) of the respondents replied that data may be misinterpreted due to complexity of the data across their research field and 71% of the respondents agree that data may be misinterpreted due to poor quality of data across their research field. Nearly three quarters (74%) of the respondents believe that data may be used in other ways than intended across their research field.

**Table 8:** Data reuse.

	Agree Strongly	Agree Somewhat	Neither Agree Nor Disagree	Disagree Somewhat	Disagree Strongly
Lack of access to data generated by other researchers or institutions is a major impediment to progress in science.	353 (27.2%)	520 (40%)	230 (17.7%)	149 (11.5%)	48 (3.7%)
Lack of access to data generated by other researchers or institutions has restricted my ability to answer scientific questions.	228 (17.6%)	422 (32.5%)	297 (22.9%)	238 (18.4%)	112 (8.6%)
Data may be misinterpreted due to complexity of the data.	383 (29.6%)	590 (45.6%)	217 (16.8%)	77 (6%)	26 (2%)
Data may be misinterpreted due to poor quality of the data.	379 (29.4%)	540 (41.8%)	232 (18%)	107 (8.3%)	33 (2.6)
Data may be used in other ways than intended.	410 (31.8%)	539 (41.8%)	249 (19.3%)	68 (5.3%)	23 (1.8%)

Respondents were asked to indicate whether they have the sole responsibility for approving access to their data. Of those who answered this question, 43% (n = 545) have the sole responsibility for all their datasets, 37% (n = 466) have for some of their datasets, and 21% (n = 266) do not have the sole responsibility.

Adding descriptive metadata to datasets helps makes the dataset more accessible by others and into the future. Respondents were asked to indicate all metadata standards they currently use to describe their data. More than half of the respondents (56%) reported that they did not use any metadata standard and about 22% of respondents indicated they used their own lab metadata standard. This could be interpreted that over 78% of survey respondents either use no metadata or a local home grown metadata approach. Clearly, educational programs including workshops and providing easy tools for metadata training could improve this situation. Awareness of why metadata improves access to data and guidance on standards would both be beneficial. The metadata standards that are used by the participants are presented in Table 9.

**Table 9:** Metadata standards.

	Responses	Percent
No metadata standard	676	56.1%
Metadata Standardized Within My Lab	266	22.1%
International Standards Organization	97	8.0%
Open GIS	96	8.0%
Ecological Metadata Language	95	7.9%
Federal Geographic Data Committee	95	7.9%
Other Metadata	82	6.8%
Dublin Core	26	2.2%
Darwin Core	21	1.7%
Directory Interchange Format	12	1.0%

#### Data Sharing

Nearly one third of the respondents chose not to answer whether they make their data available to others. Of those who did respond, 46% reported they do not make their data electronically available to others. Almost as many reported that at least some of their data are available somehow, either on their organization's website, their own website, a national network, a global network, a personal website, or other (see Table 10). The high percentage of non-respondents to this question most likely indicates that data sharing is even lower than the numbers indicate. Furthermore, the less than 6% of scientists who are making “All” of their data available via some mechanism, tends to re-enforce the lack of data sharing within the communities surveyed.

**Table 10:** Data sharing practices.

	None	Some	Most	All	Total
On My Organization's Website	495 (45.9%)	378 (35.1%)	143 (13.3%)	62 (5.8%)	**1078 (100%)**
On the Principal Investigator's Website	553 (56.7%)	303 (31.0%)	87 (8.9%)	33 (3.4%)	**976 (100%)**
Through a National Network	470 (46.4%)	331 (32.6%)	153 (15.1%)	60 (5.9%)	**1014 (100%)**
Through a Regional Network	579 (64.7%)	238 (26.6%)	58 (6.5%)	20 (2.2%)	**895 (100%)**
Through a Global Network	550 (57.6%)	242 (25.3%)	111 (11.6%)	52 (5.4%)	**955 (100%)**
On My Personal Website	668 (72.7%)	173 (18.8%)	49 (5.3%)	29 (3.2%)	**919 (100%)**
Other	370 (65.3%)	94 (16.6%)	47 (8.3%)	56 (9.9%)	**567 (100%)**

Only about a third (36%) of the respondents agree that others can access their data easily, although three-quarters share their data with others (see Table 11). This shows there is a willingness to share data, but it is difficult to achieve or is done only on request.

**Table 11:** Data sharing.

	AgreeStrongly	AgreeSomewhat	Neither Agree Nor Disagree	Disagree Somewhat	Disagree Strongly
I share my data with others.	418 (32.3%)	551 (42.6%)	199 (15.4%)	95 (7.3%)	30 (2.3%)
Others can access my data easily.	150 (11.6%)	317 (24.6%)	310 (24%)	307 (23.8%)	207 (16%)

Researchers cite many reasons why their data are not available electronically to others (see Table 12). The leading reason is insufficient time (54%), followed by lack of funding (40%). These problems are difficult to solve, but systems that make it quick and easy to share data without additional cost may help. Other reasons such as having no place to put the data (24%), lack of standards (20%), and “sponsor does not require” (17%) may be easier to resolve by subject or government initiatives or large scale projects such as DataONE and other DataNet partners. It is also important to note that only 14% of respondents stated that their data “Should not be Available”, which may bode well for the future of data sharing if logistics are resolved.

**Table 12:** Reasons for not making data electronically available.

	Responses	Percent
Insufficient Time	603	53.6%
Lack of Funding	445	39.6%
Do not Have Rights to Make Data Public	271	24.1%
No Place to Put Data	264	23.5%
Lack of Standards	222	19.8%
Sponsor does not Require	196	17.4%
Do not Need Data	169	15.0%
Other Reasons For Data Not Available	164	14.6%
Should not be Available	162	14.4%

Regarding their attitudes towards data sharing, most of the respondents (85%) are interested in using other researchers' datasets, if those datasets are easily accessible. Of course, since only half of the respondents report that they make some of their data available to others and only about a third of them (36%) report their data is easily accessible, there is a major gap evident between desire and current possibility. Seventy-eight percent of the respondents said they are willing to place at least some their data into a central data repository with no restrictions.

Data repositories need to make accommodations for varying levels of security or access restrictions. When asked whether they were willing to place all of their data into a central data repository with no restrictions, 41% of the respondents were not willing to place all of their data. Nearly two thirds of the respondents (65%) reported that they would be more likely to make their data available if they could place conditions on access.

Less than half (45%) of the respondents are satisfied with their ability to integrate data from disparate sources to address research questions, yet 81% of them are willing to share data across a broad group of researchers who use data in different ways.

Along with the ability to place some restrictions on sharing for some of their data, the most important condition for sharing their data is to receive proper citation credit when others use their data. For 92% of the respondents, it is important that their data are cited when used by other researchers. Eighty-six percent of survey respondents also noted that it is appropriate to create new datasets from shared data. Most likely, this response relates directly to the overwhelming response for citing other researchers' data. The breakdown of this section is presented in Table 13.

**Table 13:** Conditions for data sharing.

	AgreeStrongly	Agree Somewhat	Neither Agree Nor Disagree	Disagree Somewhat	Disagree Strongly
I would use other researchers' datasets if their datasets were easily accessible.	561 (43.2%)	524 (40.3%)	136 (10.5%)	62 (4.8%)	16 (1.2%)
I would be willing to place at least some of my data into a central data repository with no restrictions.	539 (41.6%)	472 (36.4%)	141 (10.9%)	104 (8%)	39 (3%)
I would be willing to place all of my data into a central data repository with no restrictions.	191 (14.9%)	338 (26.3%)	234 (18.2%)	318 (24.7%)	205 (15.9%)
I would be more likely to make my data available if I could place conditions on access.	317 (24.8%)	506 (39.6%)	279 (21.8%)	107 (8.4%)	68 (5.3%)
I am satisfied with my ability to integrate data from disparate sources to address research questions.	156 (12.2%)	419 (32.7%)	363 (28.3%)	275 (21.5%)	69 (5.4%)
I would be willing to share data across a broad group of researchers who use data in different ways.	476 (37%)	565 (43.9%)	185 (14.4%)	48 (3.7%)	13 (1%)
It is important that my data are cited when used by other researchers.	885 (68.6%)	298 (23.1%)	87 (6.7%)	14 (1.1%)	7 (0.5%)
It is appropriate to create new datasets from shared data.	505 (38.9%)	475 (36.6%)	261 (20.1%)	36 (2.8%)	20 (1.5%)

The participants were asked a series of questions about whether they find it a fair condition for the use of their data when these conditions are met. Afterwards, they were presented with the same conditions and asked whether they find each a fair condition for the use of other people's data. Respondents do not differentiate much between what they consider fair conditions for use of others' data and fair conditions for use of their own data (see Table 14). Sixty-one percent of the respondents find it fair to use other people's data if they give them co-authorship on publications resulting from use of the data. A vast majority (93%) find it a fair condition to use other people's data if there is formal acknowledgement of the data providers and/or funding agencies in all disseminated work making use of the data and 95% of the respondents reported that they find it fair to use other people's data if there is formal citation of the data providers and/or funding agencies in all disseminated work making use of the data. Also, 81% percent of the respondents reported that it is fair to use other people's data if the provider has the opportunity to collaborate on the project (including, for example, consultation on analytic methods, interpretation of results, dissemination of research results, etc.).

**Table 14:** Others using data & using others' data.

	For others to use my data		To use other people's data	
	Yes	No	Yes	No
Co-authorship on publications resulting from use of the data	751 (59.7%)	506 (40.3%)	750 (61.2%)	476 (38.8%)
Formal acknowledgement of the data providers and/or funding agencies in all disseminated work making use of the data	1168 (93%)	88 (7%)	1147 (93.3%)	83 (6.7%)
Formal citation of the data providers and/or funding agencies in all disseminated work making use of the data	1166 (94.5%)	68 (5.5%)	1152 (95.1%)	59 (4.9%)
The opportunity to collaborate on the project (including, for example, consultation on analytic methods, interpretation of results, dissemination of research results, etc.)	991 (80.6%)	239 (19.4%)	980 (81.2%)	227 (18.8%)
Results based (at least in part) on the data could not be disseminated in any format without the data provider's approval.	585 (47.7%)	642 (52.3%)	594 (48.9%)	620 (51.1%)
At least part of the costs of data acquisition, retrieval or provision must be recovered.	364 (30%)	851 (70%)	374 (31.2%)	826 (68.8%)
Results based (at least in part) on the data could not be disseminated without the data provider having the opportunity to review the results and make suggestions or comments, but approval not required.	746 (61.7%)	464 (38.3%)	750 (62.7%)	447 (37.3%)
Reprints of articles that make use of the data must be provided to the data provider.	860 (70.1%)	367 (29.9%)	850 (70.4%)	357 (29.6%)
The data provider is given a complete list of all products that make use of the data, including articles, presentations, educational materials, etc.	846 (69.3%)	375 (30.7%)	831 (69.1%)	372 (30.9%)
Legal permission for data use is obtained.	545 (44.8%)	672 (55.2%)	552 (45.8%)	652 (54.2%)
Mutual agreement on reciprocal sharing of data	880 (72.2%)	339 (27.8%)	865 (71.9%)	338 (28.1%)
The data provider is given and agrees to a statement of uses to which the data will be put.	810 (66.8%)	403 (33.2%)	799 (67%)	394 (33%)

A little more than the half (52%) of the respondents believe it is fair to disseminate results based (at least in part) on data without the data provider's approval. The respondents were asked whether it is a fair condition to use other people's data if at least part of the costs of data acquisition, retrieval or provision are recovered. Over two-thirds (69%) of them replied no, either indicating that paying for the costs of data does not include the right to use that data or that they do not believe that data users should be required to pay data creators.

Reviewing derivative works is important to many; 63% agree it is a fair condition to use other people's data if results based (at least in part) on the data are disseminated with the data provider having the opportunity to review, but not approve, the results and make suggestions or comments. In addition, 70% agree it is a fair condition to use other people's data if reprints of articles that make use of the data are provided to the data provider. Sixty-nine percent of the respondents find it fair to use other people's data if the data provider is given a complete list of all products that make use of the data, including articles, presentations, educational materials, etc. Nearly three quarters (72%) of the respondents find it fair to use other people's data if there is mutual agreement on reciprocal sharing of data.

Respondents were asked whether it is fair to use other people's data if legal permission for data use is obtained. This question is perhaps more important for researchers in corporate or other settings, where legal rights to data may be important. Slightly over half (54%) said no, indicating they feel it is not necessary or desirable to obtain legal permission. In another question, approximately two-thirds (67%) find it fair to use other people's data if the data provider is given and agrees to a statement of uses to which the data will be put.

### Demographic Groups in Relation to Data Reuse and Sharing

Not all scientists share data equally or have the same perceptions of data sharing and reuse. We found significant differences based on subject discipline, age, work focus (whether scientists are more research-focused or teaching-focused), and world region (U.S., Europe, and rest of world).

### Subject Discipline

Subject disciplines make a difference in respondents' data sharing and management practices and perceptions. Although the majority of the respondents to this survey came from the target areas of environmental, physical, or life sciences, responses also came from social sciences, engineering, medicine, and other disciplines.

#### Data use by subject discipline

A majority of all respondents report they share their data with others, but respondents from the medical fields and social sciences are less likely to make their data electronically available to others (see Table 15). Conversely, atmospheric scientists report their data is the most available to others. Of the respondents from atmospheric science, 90% report they share their data with others and 85% of biologists report they share their data. Medicine (65%), computer science/engineering (64%), and social sciences (58%) report the least amount of sharing.

**Table 15:** Conditions for data sharing by subject discipline.

	I am satisfied with my ability to integrate data from disparate sources to address research questions^1^	
	Agree strongly	Agree somewhat
social sciences	24 (11.9%)	56 (27.9%)
computer science/engineering	14 (12.3%)	35 (30.7%)
physical sciences	25 (16.8%)	45 (30.2%)
environmental sciences & ecology	53 (11.4%)	169 (36.4%)
atmospheric science	8 (16.3%)	20 (40.8%)
biology	19 (10.5%)	53 (29.3%)
medicine	1 (3.3%)	10 (33.3%)
other	12 (12.9%)	31 (33.3%)

1 *χ^2^ = 47.251, p = .013.*

Nearly three quarters (76%) of the total respondents report they share their data on their organization's website, or the PI's website or on a global network. For medicine it is around one quarter (20% to 27%) and for social sciences around one third (30% to 37%). Regional networks are the least preferred method to make data electronically available to others by all subject disciplines.

With regard to making data available, differences across disciplines in willingness to share data is part of the picture; satisfaction with current practices that make integrating other people's data is another part. Atmospheric scientists (57%) are the most satisfied with their ability to integrate data from disparate sources to address research questions, while those in medicine are the least satisfied (37%). (These results should be interpreted cautiously due to fewer respondents in these fields.)

Although a majority of respondents in every discipline report that they share their data in some way with others, most do not believe that others can access their data easily (see Table 16). Atmospheric scientists agree with the statement “others can access my data easily” in the greatest numbers (49%).

**Table 16:** Data sharing by subject discipline.

	Others can access my data easily	
	Agree strongly	Agree somewhat
social sciences	11(5.4%)	36(17.8%)
computer science/engineering	12(10.3%)	29(24.8%)
physical sciences	17(11.3%)	41(27.3%)
environmental sciences & ecology	56(12.0%)	124(26.5%)
atmospheric science	12(23.5%)	13(25.5%)
biology	28(15.6%)	50(27.9%)
medicine	2(6.5%)	2(6.5%)
other	12(13.0%)	21(22.8%)

*χ^2^ = 73.265, p = .000.*

#### Data practices by subject discipline

A majority of all respondents are satisfied with the process for collecting research data, with respondents from biology (86%) and environment science & ecology (81%) reporting the most satisfaction with the process for collecting research data. Although there are some differences, over two-thirds of the respondents in every discipline are satisfied with the process for collecting data (see Table 17).

**Table 17:** Satisfaction for data management by subject discipline.

	I am satisfied with the process for collecting my research data^1^		I am satisfied with the tools for preparing metadata^2^		I am satisfied with the tools for preparing my documentation^3^	
	Agree strongly	Agree somewhat	Agree strongly	Agree somewhat	Agree strongly	Agree somewhat
social sciences	52(25.7%)	105(52.0%)	6(3.2%)	29(15.3%)	20(10.0%)	67(33.5%)
computer science/engineering	26(22.0%)	58(49.2%)	9(7.8%)	26(22.6%)	16(13.7%)	42(35.9%)
physical sciences	51(33.1%)	71(46.1%)	9(6.3%)	35(24.5%)	21(14.1%)	49(32.9%)
environmental sciences & ecology	148(31.6%)	233(49.7%)	27(5.8%)	91(19.4%)	45(9.6%)	138(29.6%)
atmospheric science	15(30.0%)	25(50.0%)	3(6.4%)	16(34.0%)	6(12.5%)	8(16.7%)
biology	73(40.6%)	83(46.1%)	12(6.7%)	36(20.2%)	26(14.8%)	60(34.1%)
medicine	9(29.0%)	13(41.9%)	4(12.9%)	2(6.5%)	6(19.4%)	12(38.7%)
other	35(37.6%)	38(40.9%)	5(5.7%)	17(19.5%)	15(16.7%)	37(41.1%)

1 *χ^2^ = 45.210, p = .021;*

2 *χ^2^ = 47.363, p = .013;*

3 *χ^2^ = 42.346, p = .040.*

Many respondents neither agreed nor disagreed to questions about satisfaction with various tools. This is particularly true for metadata tools. The responses may indicate unfamiliarly with a function or tools for a function more than it indicates a moderate level of satisfaction. There is a statistically significant response based on subject discipline to satisfaction with two categories of tools–metadata and documentation tools. Less than half of the respondents are satisfied with the tools for preparing their documentation in all fields except medicine (58%) and computer science/engineering (50%).

#### Data management support and policies by subject discipline

There is a significant difference based on subject discipline for how respondents' organizations are involved with data. In terms of having a formal established process for managing data during the life of the project, respondents from atmospheric science (54%) and environmental sciences & ecology (48%) report the most involvement, whereas social sciences (38%) report the least.

One-half of the respondents from atmospheric science agree the most that their organizations have a formal established process for managing data beyond the life of the project. As with short-term data management during the life of the project, social sciences respondents report the lowest percentage (33%) of those who have formal, established processes for managing data beyond the life of a project. The computer science/engineering (34%), biology (35%), and medical respondents (35.5%) are also on the lower end.

Although the responses from the medical disciplines were few, a larger percent of medical respondents (55%) agree that their organization or project provides the necessary tools and technical support for data management during the life of the project, closely followed by atmospheric science (53%). Medical respondents are also more likely to agree they have the necessary tools and technical support for data management in the long-term (42% agree).

In terms of being provided the necessary funds to support data management during the life of a research project, the fewest social scientists agree (23%), with the most agreement among atmospheric scientists (42%). A vast majority of respondents in all fields say they lack the necessary funds to support data management in the long term, with only 15% of social scientists reporting sufficient funds, and the rest of the disciplines clustering between 22% and 27%.

#### Data reuse by subject discipline

Most respondents (at least 60% across disciplines) agree that lack of access to data generated by other researchers or institutions is a major impediment to progress in science. Social scientists (80%) agree at a higher rate than other respondents. Most environmental scientists & ecologists (78%) agree with the statement that data may be used in other ways than intended. The responses for data reuse by subject discipline are provided in Table 18.

**Table 18:** Data reuse by subject discipline.

	Lack of access to data generated by other researchers or institutions is a major impediment to progress in science^1^		Data may be used in other ways than intended^2^	
	Agree strongly	Agree somewhat	Agree strongly	Agree somewhat
social sciences	52(25.7%)	82(40.6%)	73(36.1%)	89(44.1%)
computer science/engineering	43(37.1%)	36(31.0%)	30(25.9%)	44(37.9%)
physical sciences	33(21.3%)	68(43.9%)	43(28.3%)	63(41.4%)
environmental sciences & ecology	128(27.1%)	203(43.0%)	158(33.7%)	207(44.1%)
atmospheric science	11(22.4%)	19(38.8%)	13(26.5%)	18(36.7%)
biology	50(27.8%)	71(39.4%)	56(31.6%)	74(41.8%)
medicine	5(16.1%)	11(35.5%)	8(25.8%)	12(38.7%)
other	30(31.9%)	30(31.9%)	29(31.5%)	32(34.8%)

1 *χ^2^ = 52.376, p = .003;*

2 *χ^2^ = 48.831, p = .009.*

#### Data sharing by subject discipline

Approximately 60% to 90% of respondents in all disciplines agree with the statement that “they would use other researchers' datasets if their datasets were easily accessible” (see Table 19). Again, a majority of respondents in almost all disciplines, (72% to 92%) “would be willing to place at least **some** of their data into a central data repository with no restrictions”(except medicine respondents with just 47%).

**Table 19:** Conditions for data sharing by subject discipline.

	I would use other researchers' datasets if their datasets were easily accessible^1^		I would be willing to place at least some of my data into a central data repository with no restrictions^2^		I would be willing to place all of my data into a central data repository with no restrictions^3^		I would be more likely to make my data available if I could place conditions on access^4^	
	Agree strongly	Agree somewhat	Agree strongly	Agree somewhat	Agree strongly	Agree somewhat	Agree strongly	Agree somewhat
social sciences	83(40.7%)	79(38.7%)	70(34.5%)	75(36.9%)	23(11.4%)	41(20.4%)	46(23.1%)	82(41.2%)
computer science/engineering	51(44.0%)	44(37.9%)	48(41.4%)	44(37.9%)	16(13.9%)	31(27.0%)	21(18.4%)	47(41.2%)
physical sciences	66(43.1%)	66(43.1%)	61(40.1%)	61(40.1%)	22(14.7%)	44(29.3%)	26(17.6%)	60(40.5%)
environmental sciences & ecology	221(47.0%)	195(41.5%)	223(47.6%)	159(34.0%)	70(15.0%)	138(29.6%)	133(28.9%)	187(40.6%)
atmospheric science	22(44.0%)	22(44.0%)	21(42.0%)	25(50.0%)	9(18.0%)	17(34.0%)	6(12.0%)	29(58.0%)
biology	67(37.0%)	74(40.9%)	75(41.4%)	68(37.6%)	39(21.7%)	39(21.7%)	56(31.1%)	53(29.4%)
medicine	8(26.7%)	10(33.3%)	4(13.3%)	10(33.3%)	1(3.3%)	4(13.3%)	8(26.7%)	11(36.7%)
other	42(44.7%)	34(36.2%)	36(38.3%)	30(31.9%)	11(11.8%)	23(24.7%)	21(22.3%)	37(39.4%)

1 *χ2 = 46.693, p = .015;*

2 *χ2 = 69.438, p = .000;*

3 *χ2 = 56.836, p = .001;*

4 *χ2 = 43.404, p = .032.*

Fewer “would be willing to place **all** of their data into a central data repository with no restrictions”. Only 41% to 52% respondents in most disciplines agree with this statement, with medicine (17%) and social sciences (32%) even less likely to agree. These disciplines are likely to have data that may be sensitive to human subject or ethical guidelines. If respondents could place conditions on access, they are more likely to make their data available. Including respondents from medicine, all values increased (between 58% and 71%). Clearly, systems for data deposit need to accommodate conditions and restrictions.

Other opinions on data sharing and data use also vary with subject discipline. Nearly all respondents in atmospheric science (94%) and environmental science & ecology (87%) say they are willing to share data across a broad group of researchers who use data in different ways. A majority of other disciplines are also willing to share, although there is a significant difference in willingness. The least willing to share among disciplines are respondents from computer science (68%) and medicine (70%).

Respondents from all disciplines (86%–100%) think it is important that their data are cited when used by other researchers. A majority of respondents (57%–83%) believe it is appropriate to create new datasets from shared data. The responses are presented in Table 20.

**Table 20:** Conditions for data sharing for reuse by subject discipline.

	I would be willing to share data across a broad group of researchers who use data in different ways^1^		It is important that my data are cited when used by other researchers^2^		It is appropriate to create new datasets from shared data^3^	
	Agree strongly	Agree somewhat	Agree strongly	Agree somewhat	Agree strongly	Agree somewhat
social sciences	60(29.7%)	100(49.5%)	119(59.5%)	55(27.5%)	77(37.9%)	73(36.0%)
computer science/engineering	36(31.9%)	41(36.3%)	68(58.6%)	32(27.6%)	40(34.5%)	36(31.0%)
physical sciences	57(38.0%)	64(42.7%)	112(74.7%)	30(20.0%)	53(34.9%)	61(40.1%)
environmental sciences & ecology	199(42.4%)	210(44.8%)	331(70.4%)	109(23.2%)	196(41.7%)	192(40.9%)
atmospheric science	18(36.7%)	28(57.1%)	40(80.0%)	10(20.0%)	17(34.0%)	19(38.0%)
biology	73(40.6%)	65(36.1%)	134(74.4%)	31(17.2%)	82(45.3%)	53(29.3%)
medicine	4(13.3%)	17(56.7%)	17(56.7%)	9(30.0%)	9(30.0%)	8(26.7%)
Other	28(30.1%)	40(43.0%)	63(67.0%)	22(23.4%)	30(31.9%)	33(35.1%)

1 *χ2 = 71.679, p = .000;*

2 *χ2 = 41.985, p = .044;*

3 *χ2 = 43.649, p = .030.*

A majority of respondents in every discipline (52%–83%) think it is a fair exchange for others to use their data if it results in co-authorship on publications resulting from use of the data (see Table 21). Most of the respondents from medicine (93%) find it a fair exchange for others to use their data if there is opportunity to collaborate on the project. For the rest of the disciplines the values are between 72% and 85%.

**Table 21:** Using others' data by subject discipline.

	Co-authorship on publications resulting from use of the data^1^	The opportunity to collaborate on the project^2^	Results based (at least in part) on the data could not be disseminated in any format without the data provider's approval^3^	At least part of the costs of data acquisition, retrieval or provision must be recovered^4^	Reprints of articles that make use of the data must be provided to the data provider^5^	Legal permission for data use is obtained^6^
social sciences	104(52.8%)	142(72.1%)	83(42.8%)	60(30.9%)	125(64.4%)	107(55.2%)
computer science/engineering	59(52.2%)	88(80.0%)	59(54.6%)	46(42.2%)	66(60.0%)	61(56.0%)
physical sciences	84(55.3%)	120(82.8%)	64(43.8%)	35(24.3%)	107(73.8%)	58(40.3%)
environmental sciences & ecology	289(63.7%)	360(80.7%)	208(46.2%)	122(27.5%)	337(75.4%)	173(38.7%)
atmospheric science	31(63.3%)	39(79.6%)	20(42.6%)	11(24.4%)	32(66.7%)	17(37.8%)
biology	105(60.7%)	143(84.6%)	84(49.7%)	42(25.3%)	114(67.1%)	65(38.9%)
medicine	24(82.8%)	27(93.1%)	20(71.4%)	15(53.6%)	22(78.6%)	19(70.4%)
other	54(60.7%)	71(84.5%)	47(56.0%)	33(38.8%)	56(66.7%)	45(54.2%)

1 *χ2 = 17.514, p = .014;*

2 *χ2 = 15.076, p = .035;*

3 *χ2 = 14.610, p = .041;*

4 *χ2 = 24.282, p = .001;*

5 *χ2 = 17.680, p = .014;*

6 *χ2 = 35.158, p = .000.*

A majority of medical respondents also agree that it is a fair exchange for others to use their data if results based (at least in part) on the data could not be disseminated in any format without the data provider's approval; it a fair exchange for others to use their data if at least part of the costs of data acquisition, retrieval or provision must be recovered; and it a fair exchange for others to use their data if legal permission for data use is obtained. Respondents in other disciplines are less likely to agree.

A majority of respondents from all fields find it a fair exchange for others to use their data if reprints of articles that make use of the data are provided to the data provider.

Results were similar for what is considered fair use of other's data (see Table 22).

**Table 22:** Using others' data by subject discipline.

	Co-authorship on publications resulting from use of the data^1^	The opportunity to collaborate on the project^2^	At least part of the costs of data acquisition, retrieval or provision must be recovered^3^	Legal permission for data use is obtained^4^
social sciences	103(54.2%)	144(73.5%)	60(30.9%)	107(55.7%)
computer science/engineering	56(51.4%)	86(77.5%)	48(44.0%)	63(58.9%)
physical sciences	83(55.7%)	119(84.4%)	40(27.8%)	60(41.4%)
environmental sciences & ecology	293(66.0%)	355(81.4%)	125(28.5%)	171(38.9%)
atmospheric science	30(63.8%)	38(79.2%)	11(25.6%)	20(42.6%)
biology	107(62.6%)	144(87.3%)	43(26.7%)	67(41.1%)
medicine	24(82.8%)	26(89.7%)	16(57.1%)	19(70.4%)
other	53(61.6%)	67(83.8%)	31(37.8%)	45(54.9%)

1 *χ2 = 20.469, p = .005;*

2 *χ2 = 15.439, p = .031;*

3 *χ2 = 23.199, p = .002;*

4 *χ2 = 35.590, p = .000.*

#### Discipline summary

Although there are significant differences in both practices and opinions about data sharing across disciplines, it is clear that data systems must accommodate restrictions or conditions for use and re-use. Most respondents are willing to share at least some of their data, if such conditions exist. Respondents in the sciences are generally more satisfied with current situations and willing to share than those in disciplines such as medicine or social sciences where human subjects or other restrictions may come into play with some datasets.

### Age

#### Data management support and policies by age

There are some differences in responses based on age of respondent. Younger people are less likely to make their data available to others (either through their organization's website, PI's website, national site, or other sites.). People over 50 showed more interest in sharing data.

There are also some differences based on the respondents' ages, in perceptions of how their organization supports data management (see Table 23). Respondents age 40–50 are less likely to agree than other age groups that their organizations have processes for managing data during the life of the project and that their organizations provide the necessary tools and technical support for data management beyond the life of the project. Younger people (ages 20–39) indicate, more than people over 40, that their organizations provide the necessary funds to support data management during and beyond the life of a research project.

**Table 23:** Organizational involvement in data issues by age group.

		Age 20–39	Age 40–50	Age over 50
My organization or project has a formal established process for managing data during the life of the project^1^	Agree Strongly	68(15.5%)	54(15.2%)	71(18.5%)
	Agree somewhat	124(28.2%)	85(23.9%)	97(25.3%)
My organization or project provides the necessary tools and technical support for data management beyond the life of the project^2^	Agree Strongly	65(14.8%)	31(8.8%)	45(11.7%)
	Agree somewhat	102(23.2%)	77(21.9%)	91(23.6%)
My organization or project provides the necessary funds to support data management during the life of a research project^3^	Agree Strongly	46(10.5%)	26(7.4%)	29(7.6%)
	Agree somewhat	106(24.1%)	78(22.1%)	68(17.8%)
My organization or project provides the necessary funds to support data management beyond the life of the project^4^	Agree Strongly	43(9.8%)	13(3.7%)	18(4.7%)
	Agree somewhat	82(18.6%)	49(14.0%)	46(11.9%)

1 *χ2 = 17.444, p = .026;*

2 *χ2 = 21.800, p = .005;*

3 *χ2 = 30.504, p = .000;*

4 *χ2 = 45.763, p = .000.*

#### Data reuse by age

Younger people were more likely to think lack of access to data is a major impediment to progress in science and has restricted their ability to answer scientific questions (see Table 24).

**Table 24:** Data reuse by age group.

		Age 20–39	Age 40–50	Age over 50
Lack of access to data generated by other researchers or institutions is a major impediment to progress in science^1^	Agree Strongly	138(30.9%)	105(29.3%)	83(21.4%)
	Agree somewhat	182(40.8%)	138(38.5%)	157(40.6%)
Lack of access to data generated by other researchers or institutions has restricted my ability to answer scientific questions^2^	Agree Strongly	100(22.4%)	63(17.6%)	46(11.9%)
	Agree somewhat	154(34.5%)	102(28.6%)	131(34.0%)

1 *χ2 = 19.082, p = .014;*

2 *χ2 = 29.320, p = .000.*

#### Data sharing by age

A majority of all respondents indicate they are not willing to place all of their data in central repositories with no restrictions, but respondents over 50 (46%) are more willing to do so than 20–39 year olds (39%) and 40–50 year olds (38%). About three-fourths of the respondents from all age groups believe it is appropriate to create new datasets from shared data. On the other hand, 20–39 year olds (69%) are slightly more likely to agree to make their data available if they could place conditions on access than 40–50 year olds (66%) and respondents over 50 (59%). The responses are provided in Table 25.

**Table 25:** Data sharing by age group.

		Age 20–39	Age 40–50	Age over 50
I would be willing to place all of my data into a central data repository with no restrictions^1^	Agree Strongly	50(11.3%)	47(13.3%)	69(18.0%)
	Agree somewhat	124(28.1%)	88(24.9%)	106(27.6%)
I would be more likely to make my data available if I could place conditions on access^2^	Agree Strongly	119(27.0%)	94(26.9%)	82(21.6%)
	Agree somewhat	187(42.4%)	135(38.6%)	143(37.7%)
It is appropriate to create new datasets from shared data^3^	Agree Strongly	175(39.1%)	132(37.0%)	161(41.7%)
	Agree somewhat	159(35.6%)	129(36.1%)	145(37.6%)

1 *χ2 = 16.072, p = .041;*

2 *χ2 = 19.507, p = .012;*

3 *χ2 = 15.620, p = .048.*

The 20–39 year old respondents are more likely to consider as fair exchange for the use of their data the provision that the data provider is given a complete list of all products that make use of the data; and legal permission for data use is obtained (see Table 26).

**Table 26:** Others using data by age group.

	The data provider is given a complete list of all products that make use of the data^1^	Legal permission for data use is obtained^2^
Age 20–39	311(74.2%)	217(51.1%)
Age 40–50	230(66.9%)	160(47.5%)
Age over 50	239(66.4%)	130(36.1%)

1 *χ2 = 7.180, p = .028;*

2 *χ2 = 18.603, p = .000.*

Younger respondents are also more likely to consider certain conditions as fair exchange for the use of other people's data, including data provider is given a complete list of all products that make use of the data (74%); legal permission for data use is obtained (72%); and data provider is given and agrees to a statement of uses to which the data will be put (72%) (see Table 27).

**Table 27:** Using others' data by age group.

	The data provider is given a complete list of all products that make use of the data^1^	Legal permission for data use is obtained^2^	The data provider is given and agrees to a statement of uses to which the data will be put^3^
Age 20–39	306(73.9%)	218(52.2%)	294(71.5%)
Age 40–50	219(65.2%)	164(49.1%)	221(66.4%)
Age over 50	241(67.5%)	131(36.6%)	224(63.3%)

1 *χ2 = 7.344, p = .025;*

2 *χ2 = 20.386, p = .000;*

3 *χ2 = 6.082, p = .048.*

Older respondents tend to have more responsibility for giving approval of data (52%). *χ^2^ = 38.912, p = .000*.

#### Age summary

Younger respondents are less likely to agree to share all of their data without restrictions, but are more likely to agree they would share some as long as restrictions are in place. Some conditions for sharing are also desirable by all age groups, but more so by those in the 20–39 age group. Receiving a complete list of products making use of their data or agreeing to that use are just some of the enticements to sharing data. Since a majority of younger scholars agree that scientific progress is inhibited by lack of access to data, providing motivations and systems for sharing may help change behavior in the future.

### Activity

Respondents were asked to report what percent of their time they spend on teaching, research, and other activities. Those who spend 50% or more of their time on teaching are recorded here as “teaching-intensive” (174 respondents), while those who spend 50% or more of their time on research are recoded as “research-intensive” (663 respondents). There are some differences between these groups in data sharing perceptions and practices.

#### Data sharing by activity

Research-intensive respondents report they are more likely to make their data available to others on organization's websites and global network than teaching-intensive respondents (see Table 28). Both research-intensive respondents (74%) and teaching-intensive respondents (79%) showed a willingness to place at least some of their data into a central data repository with no restrictions and a willingness to share data across a broad group of researchers who use data in different ways, 77% and 83% respectively. This could be attributed to data management requirements by the funding organizations.

**Table 28:** Conditions for data sharing by activity.

		Teaching-intensive	Research-intensive
I would be willing to place at least some of my data into a central data repository with no restrictions^1^	Agree Strongly	64(37.6%)	282(43.0%)
	Agree somewhat	61(35.9%)	233(35.5%)
I would be willing to share data across a broad group of researchers who use data in different ways^2^	Agree Strongly	54(32.0%)	260(39.8%)
	Agree somewhat	76(45.0%)	282(43.1%)

1 *χ2 = 11.479, p = .022;*

2 *χ2 = 12.122, p = .016.*

Research-intensive respondents (38%) agree more with the statement that “others can access my data easily” than were teaching-intensive respondents (26%) (see Table 29).

**Table 29:** Data access by activity.

	Others can access my data easily	
	Agree strongly	Agree somewhat
Teaching-intensive	14(8.3%)	30(17.9%)
Research-intensive	73(11.2%)	176(27.0%)

*χ2 = 12.270, p = .015.*

#### Data management support and policies by activity

Research-intensive respondents, compared to teaching-intensive people, are more likely to work for organizations that have processes for managing data during the life of the project (47% compared to 27%), store data beyond the life of the project (43% compared to 20%), provide the necessary tools and technical support for data management during the life of the project (49% compared to 34%), provide the necessary tools and technical support for data management beyond the life of the project (40% compared to 24%), provide training on best practices for data management (24% compared to 14%), provide the necessary funds to support data management during the life of a research project (34% compared to 18%), and provide the necessary funds to support data management beyond the life of the project (26% compared to 11%) (see Table 30).

**Table 30:** Organizational involvement by activity.

		Teaching-intensive	Research-intensive
My organization or project has a formal established process for managing data during the life of the project^1^	Agree strongly	15(8.9%)	124(19.0%)
	Agree somewhat	30(17.9%)	179(27.5%)
My organization or project has a formal established process for storing data beyond the life of the project^2^	Agree strongly	8(4.7%)	121(18.6%)
	Agree somewhat	26(15.4%)	159(24.5%)
My organization or project provides the necessary tools and technical support for data management during the life of the project^3^	Agree strongly	12(7.1%)	115(17.8%)
	Agree somewhat	46(27.1%)	203(31.4%)
My organization or project provides the necessary tools and technical support for data management beyond the life of the project^4^	Agree strongly	9(5.3%)	97(14.9%)
	Agree somewhat	31(18.3%)	162(24.9%)
My organization or project provides training on best practices for data management^5^	Agree strongly	3(1.8%)	47(7.3%)
	Agree somewhat	21(12.4%)	108(16.7%)
My organization or project provides the necessary funds to support data management during the life of a research project^6^	Agree strongly	3(1.8%)	68(10.4%)
	Agree somewhat	27(16.0%)	152(23.3%)
My organization or project provides the necessary funds to support data management beyond the life of the project^7^	Agree strongly	3(1.8%)	51(7.8%)
	Agree somewhat	16(9.4%)	121(18.6%)

1 *χ2 = 22.598, p = .000;*

2 *χ2 = 33.678, p = .000;*

3 *χ2 = 16.981, p = .002;*

4 *χ2 = 18.068, p = .001;*

5 *χ2 = 10.793, p = .029;*

6 *χ2 = 21.447, p = .000;*

7 *χ2 = 21.092, p = .000.*

#### Activity summary

Although there are fewer significant differences in practices and opinions between research-intensive and teaching-intensive respondents than there are by discipline or age, some differences exist. Research-intensive respondents are more likely to share their data and are more likely to work in organizations that support a full range of data management processes. This may be due to the type of institution they work for (a research university versus a teaching college, for example) or to the perception of those who are more focused on research or both.

### Geographic Location

Although the majority of respondents are in North America (mostly the United States, with some also from Canada), about 15% are from Europe and 12.5% from other parts of the world. There are some significant differences in some perceptions and practices among these broad regions of the world.

#### Data practices by geographic location

Researchers in North America report the most satisfaction in the process for collecting research data (82%) and storing data during the life of the project (short-term) (75%) (see Table 31).

**Table 31:** Satisfaction by geographic location.

		North American	Europe	Others
I am satisfied with the process for collecting my research data^1^	Agree strongly	311(34.0%)	44(23.8%)	47(30.1%)
	Agree somewhat	435(47.5%)	95(51.4%)	72(46.2%)
I am satisfied with the process for storing my data during the life of the project^2^	Agree strongly	279(30.7%)	47(25.7%)	41(26.6%)
	Agree somewhat	405(44.6%)	65(35.5%)	68(44.2%)

1 *χ2 = 20.009, p = .010;*

2 *χ2 = 18.201, p = .020.*

Although a majority of respondents in all regions are dissatisfied with data management processes and tools, researchers in North America showed least satisfaction with the process for storing their data beyond the life of the project (long-term) and the tools for preparing metadata, whereas researchers in Europe showed least satisfaction with the tools for preparing their documentation. Researchers in other parts of the world (non-North America) are the most satisfied with the process for storing their data beyond the life of the project (long-term), the tools for preparing metadata; and the tools for preparing their documentation (see Table 32).

**Table 32:** Satisfaction with data management by geographic location.

		North American	Europe	Others
I am satisfied with the process for storing my data beyond the life of the project^1^	Agree strongly	141(15.4%)	21(11.5%)	39(25.2%)
	Agree somewhat	251(27.5%)	59(32.2%)	45(29.0%)
I am satisfied with the tools for preparing metadata^2^	Agree strongly	39(4.4%)	12(6.7%)	21(13.9%)
	Agree somewhat	167(18.8%)	35(19.4%)	43(28.5%)
I am satisfied with the tools for preparing my documentation^3^	Agree strongly	98(10.8%)	15(8.2%)	38(24.8%)
	Agree somewhat	290(32.1%)	59(32.4%)	55(35.9%)

1 *χ2 = 24.102, p = .002;*

2 *χ2 = 34.898, p = .000;*

3 *χ2 = 36.098, p = .000.*

#### Data management support and policies by geographic location

Researchers in other (non-North America/non-Europe) parts of the world agree more than the North American respondents and European respondents that their organizations provide a formal established process for managing the data during the life of the project (Other = 51%, North American = 43%, and European = 34%); storing data beyond the life of the project (47%, 39%, and 31%); training on best practices for data management (31%, 21%, and 16%); the necessary funds to support data management during the life of a research project (39%, 31%, and 24%); and the necessary funds to support data management beyond the life of the project (32%, 22%, and 13%). Researchers in Europe showed least agreement with these statements (see Table 33).

**Table 33:** Organizational involvement in data issues by geographic location.

		North American	Europe	Others
My organization or project has a formal established process for managing data during the life of the project^1^	Agree strongly	164(17.9%)	17(9.2%)	34(22.5%)
	Agree somewhat	229(25.1%)	46(24.9%)	43(28.5%)
My organization or project has a formal established process for storing data beyond the life of the project^2^	Agree strongly	151(16.6%)	16(8.6%)	28(18.7%)
	Agree somewhat	201(22.1%)	41(22.0%)	43(28.7%)
My organization or project provides training on best practices for data management^3^	Agree strongly	48(5.3%)	5(2.7%)	19(12.8%)
	Agree somewhat	142(15.6%)	25(13.6%)	27(18.2%)
My organization or project provides the necessary funds to support data management during the life of a research project^4^	Agree strongly	87(9.5%)	9(4.8%)	17(11.4%)
	Agree somewhat	188(20.6%)	36(19.4%)	41(27.5%)
My organization or project provides the necessary funds to support data management beyond the life of the project^5^	Agree strongly	62(6.8%)	6(3.2%)	16(10.7%)
	Agree somewhat	137(15.0%)	18(9.7%)	32(21.3%)

1 *χ2 = 21.461, p = .006;*

2 *χ2 = 21.562, p = .006;*

3 *χ2 = 25.298, p = .001;*

4 *χ2 = 17.585, p = .025;*

5 *χ2 = 23.352, p = .003.*

#### Data reuse by geographical location

Researchers in other parts (non-North America/non-Europe) of the world are more likely to think that lack of access to data is a major impediment to progress in science (Other = 79%, Europe = 72%, and North America = 64%) and has restricted their ability to answer scientific questions (Other = 63%, Europe = 55%, and North America 47%. Researchers in North America were least likely to think so (see Table 34).

**Table 34:** Data reuse by geographic location.

		North America	Europe	Others
Lack of access to data generated by other researchers or institutions is a major impediment to progress in science^1^	Agree strongly	207(22.6%)	64(34.0%)	75(47.8%)
	Agree somewhat	376(41.0%)	72(38.3%)	49(31.2%)
Lack of access to data generated by other researchers or institutions has restricted my ability to answer scientific questions^2^	Agree strongly	127(13.9%)	45(23.9%)	46(29.3%)
	Agree somewhat	298(32.6%)	58(30.9%)	53(33.8%)
Data may be misinterpreted due to complexity of the data^3^	Agree strongly	276(30.3%)	57(30.5%)	40(25.6%)
	Agree somewhat	434(47.6%)	79(42.2%)	61(39.1%)
Data may be used in other ways than intended^4^	Agree strongly	312(34.3%)	42(22.5%)	46(29.5%)
	Agree somewhat	375(41.3%)	84(44.9%)	60(38.5%)

1 *χ2 = 52.125, p = .000;*

2 *χ2 = 41.971, p = .000;*

3 *χ2 = 41.022, p = .000;*

4 *χ2 = 17.484, p = .025.*

Researchers in North America showed most agreement that data may be misinterpreted due to complexity of the data (North America = 78%, Europe = 73%, and Other = 65%) and data may be used in other ways than intended (North America = 76%, Other = 68%, Europe = 67%).

Researchers in North America (84%) and Europe (84%), and other parts of the world (82%) all agree that they would use other researchers' datasets if their datasets were easily accessible.

#### Data sharing by geographic location

Researchers in other parts of the world are most willing to place all of their data into a central data repository with no restrictions (53%); more likely to make their data available if they could place conditions on access (73%); and the most satisfied with their ability to integrate data from disparate sources to address research questions (58%). Researchers in Europe (36%) showed the least support for being willing to place all of their data in a central repository with no restrictions, whereas researchers in North American are least able to integrate data from disparate sources (42%) (see Table 35).

**Table 35:** for data sharing by geographic location.

		North America	Europe	Others
I would use other researchers' datasets if their datasets were easily accessible^1^	Agree strongly	371(40.4%)	83(44.4%)	91(58.3%)
	Agree somewhat	397(43.2%)	74(39.6%)	37(23.7%)
I would be willing to place all of my data into a central data repository with no restrictions^2^	Agree strongly	127(14.0%)	23(12.4%)	36(23.5%)
	Agree somewhat	235(25.8%)	43(23.2%)	45(29.4%)
I would be more likely to make my data available if I could place conditions on access^3^	Agree strongly	201(22.3%)	54(29.0%)	52(33.8%)
	Agree somewhat	357(39.7%)	70(37.6%)	60(39.0%)
I am satisfied with my ability to integrate data from disparate sources to address research questions^4^	Agree strongly	87(9.6%)	27(14.6%)	36(23.5%)
	Agree somewhat	297(32.8%)	54(29.2%)	53(34.6%)

1 *χ2 = 28.331, p = .000;*

2 *χ2 = 24.507, p = .002;*

3 *χ2 = 17.579, p = .025;*

4 *χ2 = 42.956, p = .000.*

Non-North American/non-European, European, and North American respondents, are listed from most likely to least likely to consider certain conditions as fair exchange for the use of their data, regarding co-authorship on publications resulting from use of the data; opportunity to collaborate on the project; disseminating results based on the data with data provider's approval; recovering at least part of the costs of data acquisition, retrieval or provision; providing to the data provider the reprints of articles that make use of the data; obtaining legal permission for data use and mutual agreement on reciprocal sharing of data.

Researchers in other parts of the world, in North America, and in Europe, are from most likely to least likely to consider certain conditions as fair exchange for the use of their data, regarding formal acknowledgement of the data providers and/or funding agencies; with the data provider having the opportunity to review the results and make suggestions; giving data provider a complete list of all products that make use of the data, and giving data provider a statement of uses to which the data will be put (see Table 36).

**Table 36:** Others using data by geographic location.

	North America	Europe	Others
Co-authorship on publications resulting from use of the data^1^	504(56.9%)	112(61.5%)	113(73.4%)
Formal acknowledgement of the data providers and/or funding agencies in all disseminated work making use of the data^2^	837(93.8%)	160(88.9%)	142(94.7%)
The opportunity to collaborate on the project^3^	690(78.9%)	150(84.7%)	127(87%)
Results based (at least in part) on the data could not be disseminated in any format without the data provider's approval^4^	398(45.5%)	85(48.3%)	88(60.7%)
At least part of the costs of data acquisition, retrieval or provision must be recovered^5^	232(26.7%)	48(27.9%)	75(52.1%)
Results based (at least in part) on the data could not be disseminated without the data provider having the opportunity to review the results and make suggestions or comments, but approval not required^6^	535(61.7%)	94(55%)	98(69%)
Reprints of articles that make use of the data must be provided to the data provider^7^	593(67.8%)	125(71.4%)	118(81.4%)
The data provider is given a complete list of all products that make use of the data, including articles, presentations, educational materials, etc.^8^	593(68.2%)	109(62.3%)	121(82.9%)
Legal permission for data use is obtained^9^	347(39.9%)	88(51.2%)	93(64.1%)
Mutual agreement on reciprocal sharing of data^10^	605(69.5%)	128(73.1%)	128(89.1%)
The data provider is given and agrees to a statement of uses to which the data will be put^11^	561(64.4%)	108(64.3%)	121(84%)

1 *χ2 = 15.141, p = .001;*

2 *χ2 = 6.360, p = .042;*

3 *χ2 = 7.307, p = .026;*

4 *χ2 = 11.465, p = .003;*

5 *χ2 = 38.343, p = .000;*

6 *χ2 = 6.482, p = .039;*

7 *χ2 = 11.170, p = .004;*

8 *χ2 = 17.102, p = .000;*

9 *χ2 = 33.238, p = .000;*

10 *χ2 = 24.774, p = .000;*

11 *χ2 = 21.989, p = .000.*

Researchers in other parts of the world, Europe, and North America, are from most likely to least likely to consider certain conditions as fair exchange for the use of their data, regarding co-authorship on publications resulting from use of the data; formal citation of the data providers and/or funding agencies; opportunity to collaborate on the project; disseminating results based on the data with data provider's approval; providing to the data provider the reprints of articles that make use of the data; obtaining legal permission for data use, and mutual agreement on reciprocal sharing of data (see Table 37).

**Table 37:** Using others' data by geographic region.

	North America	Europe	Others
Co-authorship on publications resulting from use of the data^1^	509(58.4%)	114(64%)	104(72.7%)
Formal citation of the data providers and/or funding agencies in all disseminated work making use of the data^2^	818(94.3%)	166(97.1%)	140(98.6%)
The opportunity to collaborate on the project^3^	690(79.7%)	146(84.9%)	121(87.7%)
Results based (at least in part) on the data could not be disseminated in any format without the data provider's approval^4^	402(46.3%)	87(50%)	92(64.8%)
At least part of the costs of data acquisition, retrieval or provision must be recovered^5^	246(28.5%)	47(27.8%)	73(52.5%)
Reprints of articles that make use of the data must be provided to the data provider^6^	590(68.1%)	123(71.9%)	115(82.7%)
The data provider is given a complete list of all products that make use of the data, including articles, presentations, educational materials, etc.^7^	589(68.3%)	106(62%)	114(81.4%)
Legal permission for data use is obtained^8^	356(41.1%)	86(51.2%)	94(66.7%)
Mutual agreement on reciprocal sharing of data^9^	600(69.4%)	122(71.8%)	125(89.9%)
The data provider is given and agrees to a statement of uses to which the data will be put^10^	560(65%)	103(63.2%)	117(84.2%)

1 *χ2 = 11.484, p = .003;*

2 *χ2 = 6.328, p = .042;*

3 *χ2 = 6.667, p = .036;*

4 *χ2 = 16.738, p = .000;*

5 *χ2 = 33.218, p = .000;*

6 *χ2 = 12.621, p = .002;*

7 *χ2 = 14.213, p = .001;*

8 *χ2 = 34.383, p = .000;*

9 *χ2 = 25.215, p = .000;*

10 *χ2 = 21.227, p = .000.*

Researchers in North America are more likely to have sole responsibility for approving access to their data than those in Europe or other parts of the world, *χ^2^* = 13.285, p = .010. Researchers in Other parts of the world report at the time (before NSF's data management plan requirement) that they are more likely to be required to provide a data management plan than were those in North America or in Europe, *χ^2^* = 17.389, p = .002.

#### Geographic location summary

The majority of the respondents to this survey were from North America (U.S. and Canada), but results suggest some differences based on geographic location of respondents. North American respondents are the most satisfied with short-term data storage, but least satisfied with long-term data storage. Respondents outside North America and Europe report more support from their organizations for data management. All agree (to varying degrees) that there should be some conditions for sharing and re-using data. Data management is a global issue and solutions must take into account the perceptions and practices of researchers world-wide.

## Conclusion

A majority of respondents to this international survey of data practices are willing to share at least some of their data and re-use others' data pending certain conditions or restrictions on use. Getting credit through formal citation, obtaining copies of articles that use the data, and learning of products or publications that use the data are just some of the conditions that will help encourage data sharing.

Initiatives such as the DataNet partners in the United States and similar projects in other parts of the world can help build the infrastructure, policies, and best practices that will encourage data sharing. Providing a secure but flexible cyberinfrastructure while promulgating best practices such as data citation and metadata use, will help to build confidence in data sharing.

Although there is currently some satisfaction with tools for data collection and analysis, there is less awareness and satisfaction with tools for metadata creation and preservation. Most scientists do not believe their organization is doing a sufficient job in helping them achieve long-term data preservation and many researchers are not currently using international metadata standards. In addition, the results imply that there is a lack of awareness about the importance of metadata among the scientific community –at least in practice– which is a serious problem as their involvement is quite crucial in dealing with problems regarding data management. Input and training modules must be a part of systems to assist scientists with preparing their data and datasets to be retrievable into the future. Adherence to formal metadata standards is crucial to retrieval effectiveness. Moreover, the problem of easy conversion between different metadata standards needs to be addressed. Systems must support a variety of metadata standards, so the appropriate standards for different subject disciplines or types of data are addressed, but adherence to these standards needs to be easily accomplished by users who have little time to learn the specifics of metadata standards. Systems must prompt users at the time of data deposition and convert input into required standards.

The findings from this survey are both similar to and different from the PARSE Insight study done in 2009. Both studies have similar sample size (∼1300); however half of the respondents for PARSE Insight were from EU countries whereas in our study 75% of were from North America [21]. In both studies, various subject disciplines were represented, although physical sciences were the most represented in the PARSE Insight study and environmental sciences and ecology were most represented in the current study.

This study found the reasons scientists cite for not making their data electronically available to others were insufficient time and lack of funding, which is quite different from the PARSE Insight study, which found that legal issues and misuse of their data was labeled as their barrier for sharing. By packaging suites of services, perhaps efforts such as DataONE can reduce the time it takes researchers and the costs to organizations to post data.

In both our study and the PARSE Insight study, researchers reported that their ability to answer scientific questions was restricted because they could not access data generated by others. This is a serious concern since this reason is a major motivation for building a culture of data sharing, preservation, and use. Major scientific challenges of today, such as climate change and global warming can be better understood if datasets from across the sciences can be accessed and reused. Making it convenient for scientists to describe, deposit, and share their data and to access data from others, plus promulgating best data practices through education and awareness will help the future of science as well as the future of data preservation.

DataONE and similar efforts should pay close attention to organizational policies and resources. Respondents indicate satisfaction with those parts of the data lifecycle over which they can exert greater, individual control such as data collection, data description, data searching, and data documentation. These results suggest that organizations currently promote an individualized approach to science by neglecting critical practices and tools such as metadata.

Solutions and changing practices are not just a matter of time. The survey results suggest that younger scientists have special interests in protecting their data. One possible explanation may be their concerns about tenure and professional development.

Building a sound infrastructure for data sharing, preservation, and use is a challenge, but is in some ways easier than changing a culture. Subject discipline differences actually show that we are faced with multiple cultures. Researchers report many reasons why their data is not available electronically to others. The leading reasons were insufficient time and lack of funding. These are difficult to solve, but systems that make it quick and easy to share data without cost may help. Other reasons such as no place to put data, lack of standards, and sponsor does not require data sharing may be easier to resolve by federal initiatives or large scale projects such as DataONE and other DataNet partners.

## Supporting Information

Appendix S1 Survey Instrument.
(DOC)
