# Supplementary material for: Data Sharing by Scientists: Practices and Perceptions
Source: PLoS One. 2011 Jun 29;6(6):e21101. doi: 10.1371/journal.pone.0021101 (PMC3126798; doi:10.1371/journal.pone.0021101)
Supplement: Appendix S1 — Survey Instrument. (DOC) [file pone.0021101.s001.doc]

# APPENDIX S1

**SURVEY INSTRUMENT**

**Research data assessment: building an understanding of your data needs**

You are invited to participate in an NSF-sponsored research study investigating how scientists work.  Your responses will help us better understand how scientists manage their data and will contribute to building better tools and processes for data preservation.

The questionnaire should take about 15 minutes to complete.

Your participation in this research is voluntary, and you may decline to participate without risk.  While it is useful to be complete in your responses to the survey, you may skip any questions, and you are free to withdraw from the study at any time until your survey is returned (we have no way of identifying your particular responses).  We will not link your survey responses to you in any way, and we ask that you do not put any other identifying information on the survey. This will help ensure that survey responses will be anonymous.  In addition, individual responses will be kept confidential, and information from the survey will only be reported in aggregate.  As such, we do not anticipate that your participation poses any risk.

If you have any questions about the study or procedures, please contact Carol Tenopir (ctenopir@utk.edu) or Suzie Allard (sallard@utk.edu).  If you have questions about your rights as a participant, contact the Office of Research Compliance Officer at (865) 974-3466.

**By proceeding to the survey I acknowledge that I have read the above statements and that I am 18 years old or older.**


**1) What percentage of your work time is allocated to the following activities? (total to equal 100%)**

| Administration | ___________________________________ |
| --- | --- |
| Outreach | ___________________________________ |
| Policy support | ___________________________________ |
| Research | ___________________________________ |
| Teaching | ___________________________________ |
| Other | ___________________________________ |
| If you selected other, please specify: | ___________________________________ |

**2) Which one of the following best describes your primary work sector?**

 🔾 Academic
 🔾 Government
 🔾 Commercial
 🔾 Non-profit
 🔾 Other (please specify)

If you selected other, please specify ______________________________________________________________________

**3) Which one of the following best describes your primary subject discipline?**

 🔾 Atmospheric science
 🔾 Biology
 🔾 Business
 🔾 Computer science
 🔾 Ecology
 🔾 Education
 🔾 Engineering
 🔾 Environmental science
 🔾 Geology
 🔾 Hydrology
 🔾 Information science
 🔾 Law
 🔾 Medicine
 🔾 Physical sciences
 🔾 Psychology
 🔾 Social sciences
 🔾 Other (please specify)

If you selected other, please specify ______________________________________________________________________

**4) Which terms best describe the type of data you use (check all that apply)?**

 ❑ Abiotic surveys (soils, microclimate, hydrology, etc.)
 ❑ Biotic surveys
 ❑ Data models
 ❑ Experimental (involving some degree of manipulation)
 ❑ Interviews
 ❑ Observational (no manipulation involved)
 ❑ Remote-sensed abiotic data (including meteorological data)
 ❑ Remote-sensed biotic data
 ❑ Social Science Survey
 ❑ Other (please specify)

If you selected other, please specify ______________________________________________________________________

**5) If some or all of your data are available to others, these data are available:**

|  | none | some | most | all |
| --- | --- | --- | --- | --- |
| on my organization’s website | 🔾 | 🔾 | 🔾 | 🔾 |
| on the principal investigator’s website | 🔾 | 🔾 | 🔾 | 🔾 |
| through a national network | 🔾 | 🔾 | 🔾 | 🔾 |
| through a regional network | 🔾 | 🔾 | 🔾 | 🔾 |
| through a global network | 🔾 | 🔾 | 🔾 | 🔾 |
| on my personal website | 🔾 | 🔾 | 🔾 | 🔾 |
| other | 🔾 | 🔾 | 🔾 | 🔾 |

**Survey is 25 % Completed**


**6) If your data are not available electronically to others, why not (check all that apply)?**

 ❑ Lack of funding
 ❑ Lack of standards
 ❑ People don’t need them.
 ❑ There is insufficient time to make them available.
 ❑ There is no place to put them.
 ❑ They shouldn't be available.
 ❑ Sponsor doesn't require it
 ❑ Don't have the rights to make the data public
 ❑ Other (please specify)

If you selected other, please specify ______________________________________________________________________

**7) The following group of statements relates to how you collect and use research data. Tell us how much you agree with each statement using the following scale: agree strongly, agree somewhat, neither agree nor disagree, disagree somewhat, disagree strongly.**

|  | agree strongly | agree somewhat | neither agree nor disagree | disagree somewhat | disagree strongly |
| --- | --- | --- | --- | --- | --- |
| I am satisfied with the process for collecting my research data. | 🔾 | 🔾 | 🔾 | 🔾 | 🔾 |
| I am satisfied with the process for searching for my own data. | 🔾 | 🔾 | 🔾 | 🔾 | 🔾 |
| I am satisfied with the process for cataloging/describing my data. | 🔾 | 🔾 | 🔾 | 🔾 | 🔾 |
| I am satisfied with the process for storing my data during the life of the project (short-term). | 🔾 | 🔾 | 🔾 | 🔾 | 🔾 |
| I am satisfied with the process for storing my data beyond the life of the project (long-term). | 🔾 | 🔾 | 🔾 | 🔾 | 🔾 |
| I am satisfied with the process for analyzing my data. | 🔾 | 🔾 | 🔾 | 🔾 | 🔾 |
| I share my data with others. | 🔾 | 🔾 | 🔾 | 🔾 | 🔾 |
| Others can access my data easily. | 🔾 | 🔾 | 🔾 | 🔾 | 🔾 |
| I am satisfied with the tools for preparing metadata. | 🔾 | 🔾 | 🔾 | 🔾 | 🔾 |
| I am satisfied with the tools for preparing my documentation. | 🔾 | 🔾 | 🔾 | 🔾 | 🔾 |

**8) The following group of statements relates to how your organization is involved with your data. Tell us how much you agree with each using the following scale: agree strongly, agree somewhat, neither agree nor disagree, disagree somewhat, disagree strongly.**

|  | agree strongly | agree somewhat | neither agree nor disagree | disagree somewhat | disagree strongly |
| --- | --- | --- | --- | --- | --- |
| My organization or project has a formal established process for managing data during the life of the project (short-term). | 🔾 | 🔾 | 🔾 | 🔾 | 🔾 |
| My organization or project has a formal established process for storing data beyond the life of the project (long-term). | 🔾 | 🔾 | 🔾 | 🔾 | 🔾 |
| My organization or project provides the necessary tools and technical support for data management during the life of the project (short-term). | 🔾 | 🔾 | 🔾 | 🔾 | 🔾 |
| My organization or project provides the necessary tools and technical support for data management beyond the life of the project (long-term). | 🔾 | 🔾 | 🔾 | 🔾 | 🔾 |
| My organization or project provides training on best practices for data management. | 🔾 | 🔾 | 🔾 | 🔾 | 🔾 |
| My organization or project provides the necessary funds to support data management during the life of a research project (short-term). | 🔾 | 🔾 | 🔾 | 🔾 | 🔾 |
| My organization or project provides the necessary funds to support data management beyond the life of the project (long-term). | 🔾 | 🔾 | 🔾 | 🔾 | 🔾 |

**9) The following group of statements relates to your views on the use of data across your research field. Tell us how much you agree with each using the following scale: agree strongly, agree somewhat, neither agree nor disagree, disagree somewhat, disagree strongly.**

|  | agree strongly | agree somewhat | neither agree nor disagree | disagree somewhat | disagree strongly |
| --- | --- | --- | --- | --- | --- |
| Lack of access to data generated by other researchers or institutions is a major impediment to progress in science. | 🔾 | 🔾 | 🔾 | 🔾 | 🔾 |
| Lack of access to data generated by other researchers or institutions has restricted my ability to answer scientific questions. | 🔾 | 🔾 | 🔾 | 🔾 | 🔾 |
| Data may be misinterpreted due to complexity of the data. | 🔾 | 🔾 | 🔾 | 🔾 | 🔾 |
| Data may be misinterpreted due to poor quality of the data. | 🔾 | 🔾 | 🔾 | 🔾 | 🔾 |
| Data may be used in other ways than intended. | 🔾 | 🔾 | 🔾 | 🔾 | 🔾 |

**Survey is 50% Completed**


**10) The following group of statements relates to data sharing. Tell us how much you agree with each using the following scale: agree strongly, agree somewhat, neither agree nor disagree, disagree somewhat, disagree strongly.**

|  | agree strongly | agree somewhat | neither agree nor disagree | disagree somewhat | disagree strongly |
| --- | --- | --- | --- | --- | --- |
| I would use other researchers' datasets if their datasets were easily accessible. | 🔾 | 🔾 | 🔾 | 🔾 | 🔾 |
| I would be willing to place at least some of my data into a central data repository with no restrictions. | 🔾 | 🔾 | 🔾 | 🔾 | 🔾 |
| I would be willing to place all of my data into a central data repository with no restrictions. | 🔾 | 🔾 | 🔾 | 🔾 | 🔾 |
| I would be more likely to make my data available if I could place conditions on access. | 🔾 | 🔾 | 🔾 | 🔾 | 🔾 |
| I am satisfied with my ability to integrate data from disparate sources to address research questions. | 🔾 | 🔾 | 🔾 | 🔾 | 🔾 |
| I would be willing to share data across a broad group of researchers who use data in different ways. | 🔾 | 🔾 | 🔾 | 🔾 | 🔾 |
| It is important that my data are cited when used by other researchers. | 🔾 | 🔾 | 🔾 | 🔾 | 🔾 |
| It is appropriate to create new datasets from shared data. | 🔾 | 🔾 | 🔾 | 🔾 | 🔾 |

**11) Is each of the following conditions a fair exchange for the use of your data or a fair exchange for the use of other people's data?**

|  | For others to use my data | | To use other people's data | |
| --- | --- | --- | --- | --- |
|  | yes | no | yes | no |
| Co-authorship on publications resulting from use of the data | 🔾 | 🔾 | 🔾 | 🔾 |
| Formal acknowledgement of the data providers and/or funding agencies in all disseminated work making use of the data | 🔾 | 🔾 | 🔾 | 🔾 |
| Formal citation of the data providers and/or funding agencies in all disseminated work making use of the data | 🔾 | 🔾 | 🔾 | 🔾 |
| The opportunity to collaborate on the project (including, for example, consultation on analytic methods, interpretation of results, dissemination of research results, etc.) | 🔾 | 🔾 | 🔾 | 🔾 |
| Results based (at least in part) on the data could not be disseminated in any format without the data provider's approval. | 🔾 | 🔾 | 🔾 | 🔾 |
| At least part of the costs of data acquisition, retrieval or provision must be recovered. | 🔾 | 🔾 | 🔾 | 🔾 |
| Results based (at least in part) on the data could not be disseminated without the data provider having the opportunity to review the results and make suggestions or comments, but approval not required. | 🔾 | 🔾 | 🔾 | 🔾 |
| Reprints of articles that make use of the data must be provided to the data provider. | 🔾 | 🔾 | 🔾 | 🔾 |
| The data provider is given a complete list of all products that make use of the data, including articles, presentations, educational materials, etc. | 🔾 | 🔾 | 🔾 | 🔾 |
| Legal permission for data use is obtained. | 🔾 | 🔾 | 🔾 | 🔾 |
| Mutual agreement on reciprocal sharing of data | 🔾 | 🔾 | 🔾 | 🔾 |
| The data provider is given and agrees to a statement of uses to which the data will be put. | 🔾 | 🔾 | 🔾 | 🔾 |

**12) What metadata do you currently use to describe your data, if any (check all that apply)?**

 ❑ DC (Dublin Core)
 ❑ DwC (Darwin Core)
 ❑ DIF (Directory Interchange Format)
 ❑ EML (Ecological Metadata Language)
 ❑ FGDC (Federal Geographic Data Committee)
 ❑ ISO (International Standards Organization)
 ❑ OGIS (Open GIS)
 ❑ metadata standardized within my lab
 ❑ none
 ❑ Other (please specify)

If you selected other, please specify ______________________________________________________________________

**13) Do you have the sole responsibility for approving access to your data?**

 🔾 Yes - for all my datasets
 🔾 Yes - for some of my datasets
 🔾 No

**14) If no, what additional approvals would be necessary?**

____________________________________________________________________________________________________________________________________________________________________________________________________________________________

**15) Which of the following best describes your primary funding agency?**

 🔾 Federal/national government
 🔾 State/regional government
 🔾 Local government
 🔾 Corporation
 🔾 Private foundation
 🔾 Other (please specify)

If you selected other, please specify ______________________________________________________________________

**Survey is 75% Completed**


**16) My primary project funding agency requires me to provide a data management plan.**

 🔾 Yes
 🔾 No
 🔾 Don't know

**17) Which of the following do you use to access data (check all that apply)?**

 ❑ LTER (Long-tem Ecological Research Network)
 ❑ ILTER (International Long-term Ecological Research Network)
 ❑ NEON (National Ecological Observatory Network)
 ❑ NBII (National Biological Information Infrastructure)
 ❑ DAAC (A Distributed Active-Archive Center)
 ❑ GBIF (A Global Biodiversity Information Facility)
 ❑ SAEON (South African Environmental Observation Network)
 ❑ TERN (Taiwan Ecological Research Network)
 ❑ An organization-specific system
 ❑ Other (please specify)

If you selected other, please specify ______________________________________________________________________

**18) Please share any additional comments, questions, or suggestions about your use of data.**

____________________________________________________________________________________________________________________________________________________________________________________________________________________________

**19) My current position is:**

 🔾 Administrator
 🔾 Assistant Professor
 🔾 Associate Professor
 🔾 Professor
 🔾 Graduate student
 🔾 Undergraduate
 🔾 Lecturer
 🔾 Post-doctoral fellow
 🔾 Researcher
 🔾 Other (please specify)

If you selected other, please specify ______________________________________________________________________

**20) Which of the following countries is your primary place of employment?**

 🔾 Afghanistan
 🔾 Albania
 🔾 Algeria
 🔾 Andorra
 🔾 Angola
 🔾 Antigua and Barbuda
 🔾 Argentina
 🔾 Armenia
 🔾 Australia
 🔾 Austria
 🔾 Azerbaijan
 🔾 Bahamas
 🔾 Bahrain
 🔾 Bangladesh
 🔾 Barbados
 🔾 Belarus
 🔾 Belgium
 🔾 Belize
 🔾 Benin
 🔾 Bhutan
 🔾 Bolivia
 🔾 Bosnia and Herzegovina
 🔾 Botswana
 🔾 Brazil
 🔾 Brunei Darussalam
 🔾 Bulgaria
 🔾 Burkina Faso
 🔾 Burundi
 🔾 Cambodia
 🔾 Cameroon
 🔾 Canada
 🔾 Cape Verde
 🔾 Central African Republic
 🔾 Chad
 🔾 Chile
 🔾 China
 🔾 Colombia
 🔾 Comoros
 🔾 Congo
 🔾 Costa Rica
 🔾 Côte d'Ivoire
 🔾 Croatia
 🔾 Cuba
 🔾 Cyprus
 🔾 Czech Republic
 🔾 Democratic People's Republic of Korea
 🔾 Democratic Republic of the Congo
 🔾 Denmark
 🔾 Djibouti
 🔾 Dominica
 🔾 Dominican Republic
 🔾 Ecuador
 🔾 Egypt
 🔾 El Salvador
 🔾 Equatorial Guinea
 🔾 Eritrea
 🔾 Estonia
 🔾 Ethiopia
 🔾 Fiji
 🔾 Finland
 🔾 France
 🔾 Gabon
 🔾 Gambia
 🔾 Georgia
 🔾 Germany
 🔾 Ghana
 🔾 Greece
 🔾 Grenada
 🔾 Guatemala
 🔾 Guinea
 🔾 Guinea-Bissau
 🔾 Guyana
 🔾 Haiti
 🔾 Honduras
 🔾 Hungary
 🔾 Iceland
 🔾 India
 🔾 Indonesia
 🔾 Iran (Islamic Republic of)
 🔾 Iraq
 🔾 Ireland
 🔾 Israel
 🔾 Italy
 🔾 Jamaica
 🔾 Japan
 🔾 Jordan
 🔾 Kazakhstan
 🔾 Kenya
 🔾 Kuwait
 🔾 Kyrgyzstan
 🔾 Lao People's Democratic Republic
 🔾 Latvia
 🔾 Lebanon
 🔾 Lesotho
 🔾 Liberia
 🔾 Libyan Arab Jamahiriya
 🔾 Liechtenstein
 🔾 Lithuania
 🔾 Luxembourg
 🔾 Madagascar
 🔾 Malawi
 🔾 Malaysia
 🔾 Maldives
 🔾 Mali
 🔾 Malta
 🔾 Marshall Islands
 🔾 Mauritania
 🔾 Mauritius
 🔾 Mexico
 🔾 Micronesia (Federated States of)
 🔾 Monaco
 🔾 Mongolia
 🔾 Morocco
 🔾 Mozambique
 🔾 Myanmar
 🔾 Namibia
 🔾 Nepal
 🔾 Netherlands
 🔾 New Zealand
 🔾 Nicaragua
 🔾 Niger
 🔾 Nigeria
 🔾 Norway
 🔾 Oman
 🔾 Pakistan
 🔾 Palau
 🔾 Panama
 🔾 Papua New Guinea
 🔾 Paraguay
 🔾 Peru
 🔾 Philippines
 🔾 Poland
 🔾 Portugal
 🔾 Qatar
 🔾 Republic of Korea
 🔾 Republic of Moldova
 🔾 Romania
 🔾 Russian Federation
 🔾 Rwanda
 🔾 Saint Kitts and Nevis
 🔾 Saint Lucia
 🔾 Saint Vincent and the Grenadines
 🔾 Samoa
 🔾 San Marino
 🔾 Sao Tome and Principe
 🔾 Saudi Arabia
 🔾 Senegal
 🔾 Seychelles
 🔾 Sierra Leone
 🔾 Singapore
 🔾 Slovakia
 🔾 Slovenia
 🔾 Solomon Islands
 🔾 Somalia
 🔾 South Africa
 🔾 Spain
 🔾 Sri Lanka
 🔾 Sudan
 🔾 Suriname
 🔾 Swaziland
 🔾 Sweden
 🔾 Switzerland
 🔾 Syrian Arab Republic
 🔾 Taiwan
 🔾 Tajikistan
 🔾 Thailand
 🔾 Togo
 🔾 Trinidad and Tobago
 🔾 Tunisia
 🔾 Turkey
 🔾 Turkmenistan
 🔾 Uganda
 🔾 Ukraine
 🔾 United Arab Emirates
 🔾 United Kingdom of Great Britain and Northern Ireland
 🔾 United Republic of Tanzania
 🔾 United States of America
 🔾 Uruguay
 🔾 Uzbekistan
 🔾 Vanuatu
 🔾 Venezuela
 🔾 Viet Nam
 🔾 Yemen
 🔾 Yugoslavia
 🔾 Zambia
 🔾 Zimbabwe

**21) If your response to question #20 was "United States of America," also indicate your primary state (or US territory) of employment.**

 🔾 Alabama
 🔾 Alaska
 🔾 Arizona
 🔾 Arkansas
 🔾 California
 🔾 Colorado
 🔾 Connecticut
 🔾 Delaware
 🔾 D.C.
 🔾 Florida
 🔾 Georgia
 🔾 Hawaii
 🔾 Idaho
 🔾 Illinois
 🔾 Indiana
 🔾 Iowa
 🔾 Kansas
 🔾 Kentucky
 🔾 Louisiana
 🔾 Maine
 🔾 Maryland
 🔾 Massachusetts
 🔾 Michigan
 🔾 Minnesota
 🔾 Mississippi
 🔾 Missouri
 🔾 Montana
 🔾 Nebraska
 🔾 Nevada
 🔾 New Hampshire
 🔾 New Jersey
 🔾 New Mexico
 🔾 New York
 🔾 North Carolina
 🔾 North Dakota
 🔾 Ohio
 🔾 Oklahoma
 🔾 Oregon
 🔾 Pennsylvania
 🔾 Rhode Island
 🔾 South Carolina
 🔾 South Dakota
 🔾 Tennessee
 🔾 Texas
 🔾 Utah
 🔾 Vermont
 🔾 Virginia
 🔾 Washington
 🔾 West Virginia
 🔾 Wisconsin
 🔾 Wyoming

**22) What is your age?**

 ____________________________________________________________

**23) Gender**

 🔾 Male
 🔾 Female


**Your survey is now complete.**
Thank you very much!
